# Synthesis, Properties and Spatial Structure of 4-[(3,5-Dimethyl-1,2-oxazol-4-yl)sulfonyl]cytisine

**DOI:** 10.3390/plants12010137

**Published:** 2022-12-27

**Authors:** Marat K. Ibrayev, Oralgazy A. Nurkenov, Zhanara B. Rakhimberlinova, Altynaray T. Takibayeva, Dastan M. Turdybekov, Tulegen M. Seilkhanov, Meruyert B. Issabayeva, Assel A. Kelmyalene, Assel T. Kezdikbayeva, Anel Z. Mendibayeva

**Affiliations:** 1 Abylkas Saginov Karaganda Technical University, Ave. N. Nazarbayev, 56, Karaganda 100027, Kazakhstan; 2 Faculty of Chemistry, Karaganda Buketov University, st. University 28, Karaganda 100024, Kazakhstan; 3 Institute of Organic Synthesis and Coal Chemistry of Republic of Kazakhstan, st. Alikhanov, 1, Karaganda 100008, Kazakhstan; 4 Laboratory of Engineering Profile NMR Spectroscopy, Sh. Ualikhanov Kokshetau University, Kokshetau 020008, Kazakhstan; 5 School of Pharmacy, Medical University of Karaganda, Karaganda 100008, Kazakhstan

**Keywords:** sulfo-derivatives of azoles, alkaloid cytisine, spatial structure, hemorheological activity, ^1^H, ^13^C, 2D NMR spectroscopy, X-ray diffraction analysis

## Abstract

This article has studied the synthesis of a new derivative of the known alkaloid cytisine contained in the seeds of plants of Cytisus laburnum L. and Thermopsis lanceolata R.Br., both of the Lugiminosae family. The new compound has been obtained from two biologically active compounds, such as isoxazole and cytisine. It has been demonstrated that the reaction led to the single-stage method under very mild conditions to obtain 4-[(3,5-dimethyl-1,2-oxazol-4-yl)sulfonyl]cytisine. This class of compounds is promising for obtaining the new biologically active compounds. This article has examined, in detail, a structure with using the ^1^H and ^13^C NMR and two-dimensional NMR spectroscopy of COSY (^1^H-^1^H), HMQC (^1^H-^13^C) and HMBC (^1^H-^13^C). As a result, the homo- and heteronuclear spin-spin couplings should be established. The X-ray diffraction analysis has determined the spatial structure of a new derivative based on the cytisine alkaloid. Thus, its hemorheological activity has been studied.

## 1. Introduction

Sulfo-derivatives of azoles have been widely used in pharmacy as biologically active compounds [1]. Noteworthy among them is a group of nonsteroidal anti-inflammatory drugs such as coxibs. They inhibit cyclooxygenase [2]. The study of the biological activity of azole-containing systems has included a group of sulfonamides. Their representatives had some sulfonamide derivatives of phenylisoxazole, such as sulfamethoxazole [3], sulfafurazole [4], sulfamoxole [5] and sulfafenazole, used in veterinary medicine [6]. Sulfonamides are antibacterial agents that inhibit the growth of gram-negative and gram-positive bacteria.

Addition of a sulfonamide fragment into a natural compound’s molecule, in particular an alkaloid cytisine, is of particular interest, i.e., the obtained compounds inhibit human carbonic anhydrase. The study of carbonic anhydrase inhibitors is a rapidly developing area in medicinal chemistry. Thus, carbonic anhydrase plays a key role in some biochemical processes based on the physiology of the pathological states [7,8].

## 2. Results and Discussion

This study has used 3,5-dimethylisoxazole, which has a wide spectrum of biological activity. Some publications have described the physiological activity of compounds derived from sulfo-derivatives of 3,5-dimethylisoxazole [9,10,11,12]. The published results have demonstrated that developing the synthesis methods for the new isoxazole derivatives was an urgent task in medicinal chemistry. Therefore, modification of the natural alkaloid cytisine with the addition of an isoxazole molecule (or a substituted isoxazole) of a sulfogroup is one of the advancing directions in the search for the new biologically active compounds.

The 3,5-dimethylisoxazole has been sulfochlorinated, as described in [13]. Two methyl groups in a 3,5-dimethylisoxazole molecule have increased the electron density on the ring. Thus, it has intensified the probability of sulfochlorination at position 4.

**Scheme 1:**
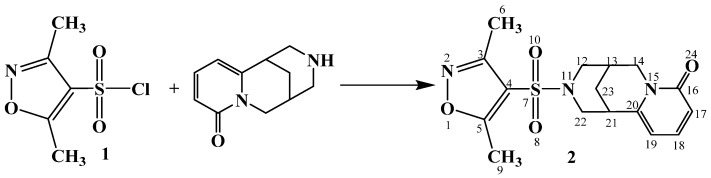
Synthesis of 4-[(3,5-dimethyl-1,2-oxazol-4-yl)sulfonyl]cytisine.

The 4-[(3,5-dimethyl-1,2-oxazol-4-yl)sulfonyl]cytisine **2** has been obtained by reacting a sulfochloride **1** with cytisine in the presence of a pyridine base (Scheme 1).

Sulfamide **2** has been synthesized by the reaction of sulfochloride **1** with cytisine in dry acetonitrile in the presence of pyridine as an acid-binding agent. The structure of 4-[(3,5-dimethyl-1,2-oxazol-4-yl)sulfonyl]cytisine **2** has been proven with ^1^H NMR spectroscopy (Figure S1, Supporting Information) and mass spectrometry (MS) (Figure S11, Supporting Information).

The method has been used because the studied sulfamide **2** had some diverse and characteristic protons, and their signals could be easily identified in the ^1^H NMR spectrum.

Thus, the ^1^H NMR spectrum of compound **2** had the protons of the bispidine cycles of cytisine fragment at 1.76–1.85 m (2H, H-23ax, 23eq), 2.49 s (4H, H-9,9,9,13), 2.83 d (1H, H-12ax, ^2^J 11.4 Hz), 2.92 d (1H, H-12eq, ^2^J 11.4 Hz), 3.22 s (1H, H-21), 3.56 d (1H, H-22ax, ^2^J 10.8 Hz), 3.61 d (1H, H-22eq, ^2^J 10.8 Hz), and 3.73–3.83 m (2H, H-14ax,14eq) ppm. The aromatic protons of a cytisine group have been recorded at 6.30 br. s (1H, H-17), 6.36 d (1H, H-19, ^3^J 9.2 Hz), and 7.43 t (1H, H-18, ^2^J 6.6 Hz) ppm. The methyl protons of H-6,6,6 and H-9,9,9 of an isoxazole fragment have been observed as triplet singlets at 2.12 ppm and 2.49 ppm, respectively.

The ^13^C NMR spectrum of compound **2** has demonstrated the signals of carbon atoms in a cytisine fragment at 24.07 (C-23), 26.59 (C-13), 33.60 (C-21), 49.70 (C-14), 51.20 (C-22), C-12), 106.91 (C-19), 115.90 (C-17), 140.37 (C-18), 150.60 (C-20) and 162.45 (C-16) ppm. The carbon atoms of a dimethylisoxazole fragment have been recorded at 11.04 (C-6), 12.95 (C-9), 113.30 (C-4), 158.21 (C-3) and 174.45 (C-5) ppm.

A structure of compound **2** has been confirmed with 2D NMR spectroscopy of COSY (^1^H-^1^H), HMQC (^1^H-^13^C) and HMBC (^1^H-^13^C). Thus, the homo- and hetero-nuclear spin-spin coupling has been established (Figures S2–S5, Supporting Information).

**Figure 1:**
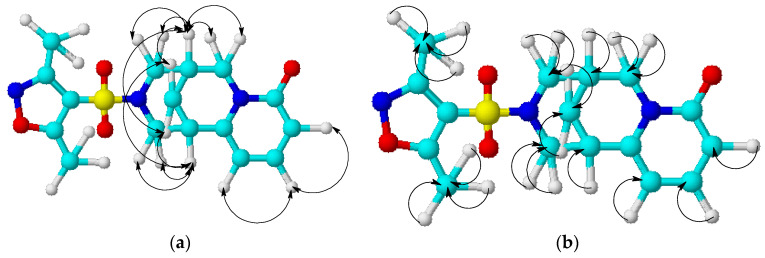
The structural correlations in COSY (**a**) and HMQC (**b**) spectra of compound **2**.

The observed NMR correlations of COSY (^1^H-^1^H) and HMQC (^1^H-^13^C) in a molecule are illustrated in Figure 1.

The ^1^H-^1^H COSY spectra of compound **2** have demonstrated the spin-spin correlations through three proton bonds of near methylene-methylene, methine-methylene and methine-methine groups of H^23^-H^21^ (1.80, 3.21 and 3.21, 1.80), H^13^-H^14^ (2.50, 3.76 and 3.76, 2.50), H^12ax^-H^22eq^ (2.81, 3.60 and 3.60, 2.81), H^12eq^-H^22ax^ (2.90, 3.49 and 3.49, 3.49), H^19^-H^18^ (6.28, 7.42 and 7.42. 6.28) and H^17^-H^18^ (6.31, 7.41 and 7.41, 6.31) ppm.

Hetero-nuclear couplings of protons with carbon atoms through a single bond have been established by ^1^H-^13^C HMQC spectroscopy for the following pairs in a compound: H^6^-C^6^ (2.10, 11.37), H^9^-C^9^ (2.47, 12.85), H^23^-C^23^ (1.79, 24.74), H^13^-C^13^ (2.50, 26.87), H^21^-C^21^ (3.20, 33.83), H^12ax^-C^12^ (2.81, 52.89), H^12eq^-C^12^ (2.93, 52.44), H^22ax^-C^22^ (3.46, 51.14),H^22eq^-C^22^ (3.60, 51.38), H^14^-C^14^ (3.76, 50.01), H^19^-C^19^ (6.28, 107.35), H^17^-C^17^ (6.34, 116.27) and H^18^-C^18^ (7.42, 140.69) ppm.

Hetero-nuclear couplings of protons with carbon atoms through two or more bonds have been determined by ^1^H-^13^C HMBC spectroscopy for the following pairs in a compound: H^19^-C^17^ (6.28, 116.38), H^19^-C^20^ (6.28, 150.54); H^9^-C^4^ (2.47, 114.10), H^9^-C^3^ (2.47, 157.09), H^9^-C^5^ (2.47, 175.02); H^6^-C^4^ (2.10, 114.11), H^6^-C^3^ (2.10, 158.22) and H^23^-C^20^ (1.78, 150.54) ppm.

**Figure 2:**
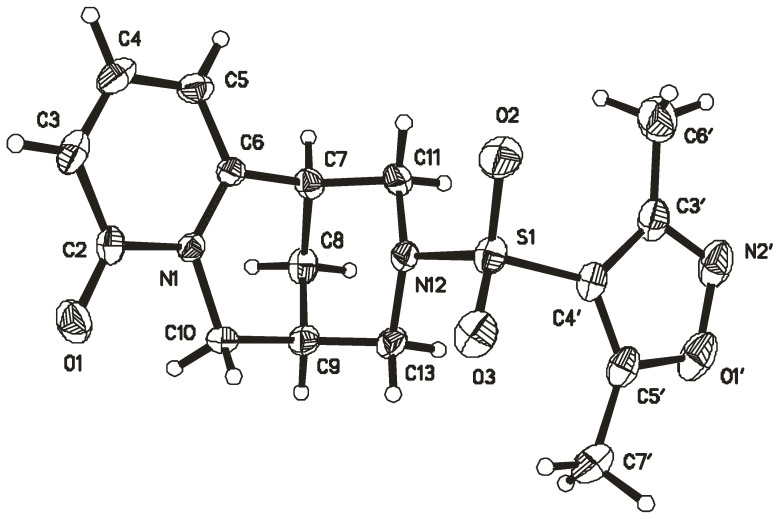
A general view of molecule **2**.

In order to establish the spatial structure of the obtained 4-[(3,5-dimethyl-1,2-oxazol-4-yl)sulfonyl]cytisin **2**, its X-ray diffraction analysis has been performed (Cif-file and Tables S1–S3, Supporting Information). A general view of a molecule is illustrated in Figure 2.

An absolute configuration has been reliably defined. The configuration of the C7 and C9 chiral centers has been correlated with the known quantity in the (−)-cytisine molecule [14]. Flack parameter has been 0.01(9) [15].

The bond lengths (Table 1) and bond angles (Table 2) in a cytisine skeleton of structure **2** have been found to be standard [16], except for the bond angles of the N12 atom (Table 2).

A dihydropyridine cycle has been flat at ±0.007 Å. A carbonyl O1 atom has almost been in the plane in atoms of this cycle. The deviation has been 0.03 Å.

The N1C6C7C8C9C10 tetrahydropyridine cycle has been in a distorted 8α-chair conformation (ΔC_S_^8^ = 3.02°). A bridging C8 atom has been observed from the middle plane of the remaining atoms (±0.01 Å) of the cycle by 0.73 Å. A piperidine cycle has been in a distorted chair conformation (ΔC_S_^8^ = 0.99°, ΔC_2_^7,8^ = 1.67°). An isoxazole cycle has been planar and its accuracy ±0.002 Å. The methyl groups have been found in a plane of the five-membered cycle. The values of torsion angles are presented in Table 3.

The configuration of the nitrogen atom (N12) in a pyridine cycle of molecule **2** has been observed to be intermediate between pyramidal and trigonal planar. The sum of the bond angles has been 345.9°. It has been caused by a partial interaction between a lone electron pair of nitrogen atom N12 and π-electrons of S=O bonds. A pyramidal configuration of nitrogen atoms has been found in the N-derivatives of cytosine. As a result, the addition of any groups has not led to the conjugation of a nitrogen lone electron pair with electrons from these groups. Thus, the electronic configuration of the N12 atom has been pyramidal in molecules of nitromethylcytisine [17] and cytisine [18] without the corresponding conjugation. The sum of bond angles has been 333.7 and 338.3°, respectively. Here and below, the data on the crystal structures have been taken from the Cambridge Structural Database [19].

Various steric strain groups have been found in cytisine derivatives conjugated with a nitrogen lone electron pair. Thus, atom configuration has changed, and it had a trigonal planar form, e.g., in molecules of ammonium N-cytisine dithiocarbamate crystal hydrate [20] and N-formylcytisine [21]. It has been explained by a mesomeric effect with conjugation between the nitrogen lone electron pair and the S-C-S (C=O) bond. Table 4 demonstrates the atomic coordinates of a structure in cell fractions of compound **2**.

**Scheme 2:**
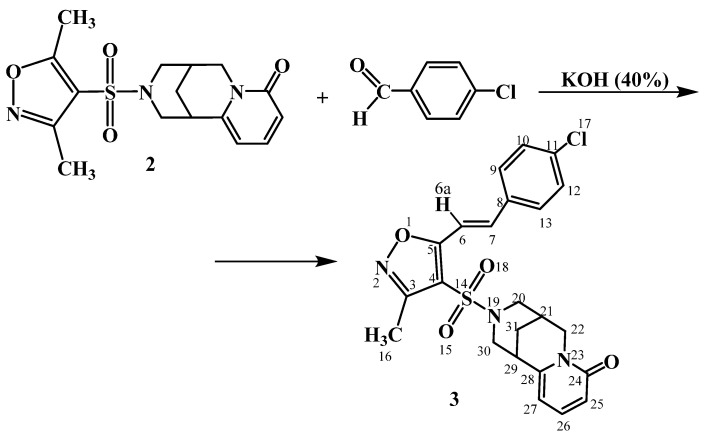
Synthesis of 4-[(3-methyl-5-{(4-chlorophenyl)ethenyl}-1,2-oxazol-4-yl)sulfonyl]cytisine.

To synthesize the sulfonamide derivatives of 3-methyl-5-vinyl-substituted isoxazoles, a method has been developed [22]. It has been based on the preliminary functionalization of the 3,5-dimethylisoxazole molecule. The reaction of sulfochlorination and amination with cytosine has been performed. As a result, a methyl group has been activated at the 5-position, and a vinyl fragment has been constructed. The 4-[(3,5-dimethyl-1,2-oxazol-4-yl)sulfonyl]cytisine **2** with its electron-accepting properties has demonstrated that a sulfamide group at the 4-position of the isoxazole cycle has increased the acidity of methyl groups at the 3- and 5-positions. In order to establish the preferential electron-accepting effect of a sulfogroup on a methyl group at the 3- or 5-positions, a condensation reaction has been performed with *p*-chlorobenzaldehyde. As a result, a methyl group at the 5-position of an isoxazole cycle has been more active. Thus, a condensation product by a methyl group at the 5-position of **3** has been obtained. A reaction has occurred with strong bases and potassium hydroxide (Scheme 2). The mixture has been heated for 1 h at 60 °C. The regioselectivity of the reaction has been established with a combination of NMR spectroscopy methods (Figure S6, Supporting Information) and mass spectrometry (MS) (Figure S12, Supporting Information).

The ^1^H NMR spectrum of compound **3** has been characterized by protons of the bispidine cycles of a cytisine fragment at 1.74–1.77 m (2H, H-31ax, 31eq), 2.45 s (1H, H-21), 2.81 d (1H, H-20ax, ^2^J 11.6 Hz), 2.91 d (1H, H-20eq, ^2^J 11.6 Hz), 3.17 s (1H, H-29), 3.29–3.83 m (H-22ax, 22eq, 30ax, 30eq) ppm. The aromatic protons of a cytisine group have been recorded at 6.15–6.17 m (1H, H-27), 6.22–6.24 m (1H, H-25) and 7.30–7.33 m (1H, H-26) ppm.

The methyl protons of the H-16,16,16 oxazole fragment have been observed as a three-proton singlet in a strong-field region of the spectrum at 2.5 ppm. Thus, it has been at the 5-position at 2.50 ppm. The aromatic protons of a chlorophenyl fragment of H-10, 12 and H-9, 13 has been resonated with two-proton multiplets at 7.46–7.49 and 7.69–7.71 ppm, respectively. For protons of a vinyl fragment at the 3-position of an isoxazole cycle, the spectrum has been characterized with two doublet signals at 7.19 ppm (H-6) and 7.63 ppm (H-7). The high spin-spin coupling constant has been 16.5 Hz. Hence, an ethylene fragment in the form of an E-diastereomer has been observed.

The ^13^C NMR spectrum of compound **3** has demonstrated the signals of carbon atoms of a cytisine fragment at 24.12 (C-31), 26.75 (C-21), 33.87 (C-29), 49.27 (C-30), 51.07 (C-22), 52.80 (C-20), 105.65 (C-27), 117.13 (C-25), 140.08 (C-26), 150.11 (C-28) and 162.62 (C-24) ppm. The carbon atoms of a methylchlorophenyloxazole fragment have been recorded at 13.01 (C-16), 112.57 (C-4), 129.58 (C-6), 130.28 and 131.64 (C-9, 10, 12, 13), 133.96 (C-11), 138.52 (C-7, 8), 150.11 (C-5) and 158.52 (C-3) ppm.

A structure of compound **3** has been confirmed by 2D NMR spectroscopy of COSY (^1^H-^1^H), HMQC (^1^H-^13^C) and HMBC (^1^H-^13^C) (Figures S7–S10, Supporting Information).

**Figure 3:**
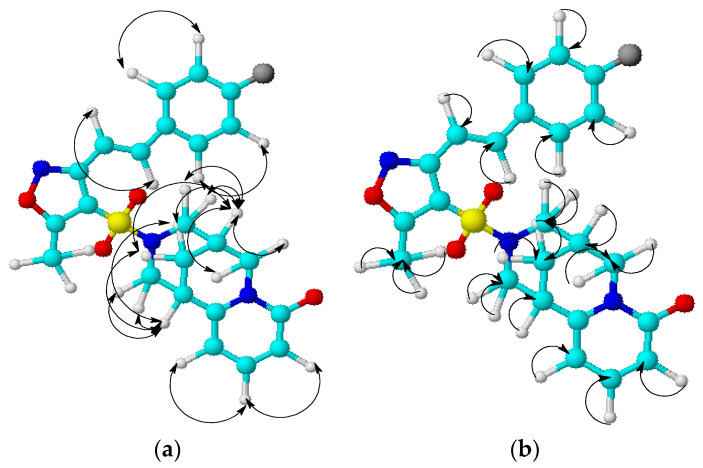
The structural correlations in the COSY (**a**) and HMQC (**b**) spectra of compound **3**.

The observed NMR correlations of COSY (^1^H-^1^H) and HMQC (^1^H-^13^C) in a molecule are illustrated in Figure 3.

The ^1^H-^1^H COSY spectra of compound **3** have been as follows: of H^31^-H^29^ (1.76, 3.16 and 3.16, 1.76), H^20eq^-H^30eq^ (2.90, 3.58 and 3.58, 2.90), H^20eq^-H^30ax^ (2.88, 3.49 and 3.49, 2.88), H^25^-H^26^ (6.21, 7.33 and 7.33, 6.21), H^27^-H^26^ (6.16, 7.32 and 7.32, 6.16), H^6^-H^7^ (7.20, 7.63 and 7.63, 7.20) and H^10,12^-H^9,13^ (7.46, 7.70 and 7.70, 7.46) ppm.

Hetero-nuclear couplings of protons with carbon atoms through a single bond have been established by ^1^H-^13^C HMQC spectroscopy for the following pairs in a compound: H^31^-C^31^ (1.76, 24.48), H^21^-C^21^ (2.8, 26.78), H^16^-C^16^ (2.49, 13.01), H^20ax^-C^20^ (2.78, 52.03), H^20eq^-C^20^ (2.90, 51.61), H^22ax^-C^22^ (3.53, 51.61), H^22eq^-C^22^ (3.70, 51.61), H^29^-C^29^ (3.16, 33.87), H^30ax^-C^30^ (3.49, 49.81), H^30eq^-C^30^ (3.60, 50.20), H^26^-C^26^ (7.31, 140.08), H^7^-C^7^ (7.63, 138.62), H^6^-C^6^ (7.20, 128.82), H^25^-C^25^ (6.22, 117.13), H^27^-C^27^ (6.16, 105.65), H^10,12^-C^10,12^ (7.45, 130.07) and H^9,13^-C^9,13^ (7.69, 130.28) ppm.

Hetero-nuclear couplings of protons with carbon atoms through two or more bonds have been determined by ^1^H-^13^C HMBC spectroscopy for the following pairs in a compound: H^16^-C^4^ (2.49, 113.39) ppm.

An in vitro biological screening has been performed for the hemorheological activity of compound **2**.

*Hemorheological activity*

The experiments on the hemorheological activity of sample **2** have found that blood incubation for 60 min at a temperature of 43.0 °C has led to a significant increase in blood viscosity at various spindle speeds from 2 s^−1^ to 60 s^−1^. Hence, the formation of blood hyperviscosity has been observed [23].

**Figure 4:**
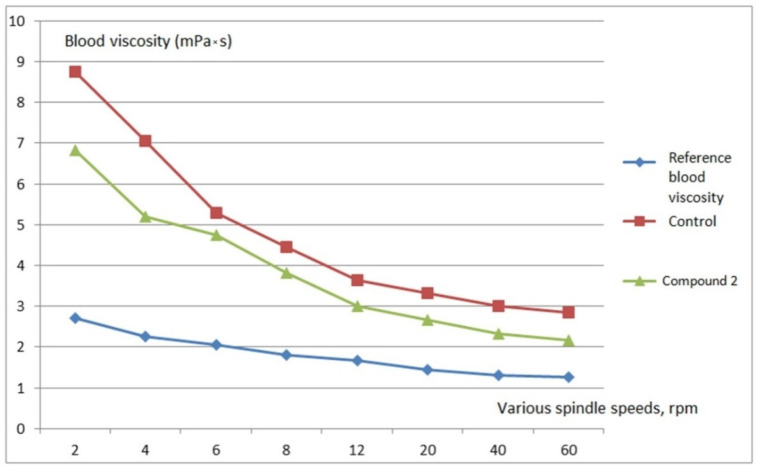
The hemorheological activity of compound **2**.

Table 5 and Figure 4 have quoted the screening results of compound **2** for the hemorheological activity in the in vitro model of blood hyperviscosity.

**Figure 5:**
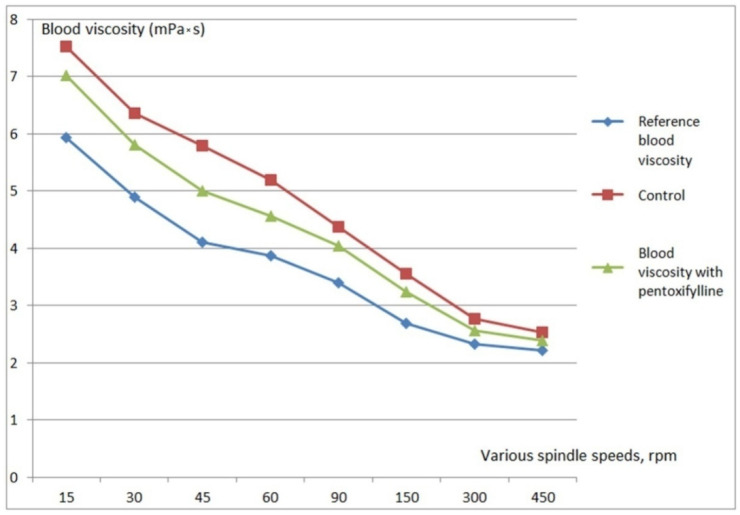
The hemorheological activity of pentoxifylline.

In order to perform a comparative analysis on a hemorheological activity, similar experiments have been made with a reference drug such as an angioprotector pentoxifylline. Results of these experiments are listed in Table 6 and Figure 5.

Thus, the obtained experimental data has demonstrated the following result of the in vitro biological screening for hemorheological activity that compound 2 has been able to reduce blood viscosity in the in vitro hyperviscosity model, and it has been as good as the reference drug pentoxifylline in the hemorheological effects in the in vitro hyperviscosity model.

## 3. Conclusions

As a result of this study, a new compound, 4-[(3,5-dimethyl-1,2-oxazol-4-yl)sulfonyl]cytisin 2, has been synthesized and has been comprehensively investigated. Its structure has contained an isoxazole heterocycle, a sulfogroup and an alkaloid cytisine.

1. Structures of compounds 2 and 3 have been determined with ^1^H, ^13^C NMR spectroscopy.
2. 2D NMR spectroscopy of COSY (^1^H-^1^H), HMQC (^1^H-^13^C) and HMBC (^1^H-^13^C) has been used to study the mutual influence of atoms inside molecules of 2 and 3.
3. The X-ray diffraction analysis has established the spatial structure of compound 2. All the parameters of a crystal structure and its structural features have been determined.
4. The reaction with p-chlorobenzaldehyde has defined that a methyl group at the 5-position of the 4-sulfamidisoxazole cycle has been more active in the condensation reaction with the substituted aromatic aldehydes.
5. The biological screening has shown a high hemorheological activity of 4-[(3,5-dimethyl-1,2-oxazol-4-yl)sulfonyl]cytisine 2. It has been as good as the well-known angioprotector pentoxifylline.

## 4. Experimental

^1^H and ^13^C NMR spectra have been taken on the JNM-ECA Jeol 400 spectrometer (JEOL Ltd. Japan, Tokyo; frequencies of 399.78 and 100.53 MHz, respectively) with the use of CDCl_3_ solvent. The chemical shifts have been measured relative to signals of residual protons or carbon atoms in deuterated chloroform. A melting point of a substance has been determined on the SMP10 device. The reaction and purity of an obtained compound have been monitored with thin-layer chromatography on Silufol UV-254 plates in the system of isopropyl alcohol-benzene-25% ammonia solution, 10:5:2. The plates have been exposed to iodine vapor.

The X-ray diffraction experiment. The cell parameters and intensities of 8569 reflections (3147 independent, R_int_ = 0.029) have been measured on a Bruker KARRA APEX2 CCD (MoK_α_; Bruker corp. Germany, Karlsruhe) diffractometer, graphite monochromator, φ, θ-scanning, 2.721° < θ < 25.990°) at a temperature of 296 K.

Crystals **2** was monoclinic, a space group P2_1_, a = 6.6291(3) Å, b = 8.9064(5) Å, c = 13.9604(7) Å, β = 98.093(2)°, V= 816.03(7) A^3^, Z = 2 (C_16_H_19_N_3_O_4_S), M = 349.40 g/mol, d_calc._ = 1.422 g/cm^3^, µ = 0.225 mm^−1^. The array of measured intensities has been processed. Absorption corrections have been performed using the SAINT [24] and SADABS [25] programs included in the APEX2 software package (multiscan, T_min_ = 0.871, T_max_ = 0.904).

The structure has been decoded with a direct method. The positions of the non-hydrogen atoms have been clarified in the anisotropic approximation by the full matrix MNA. Hydrogen atoms have been placed in geometrically calculated positions. Their positions have been refined in the isotropic approximation with fixed positional and thermal parameters (a “rider” model). The calculations have used 2944 independent reflections with I > 2σ(I). The number of the refined parameters is 219. Final divergence factors have been R1 = 0.0317, wR_2_ = 0.0749 for pictures with I > 2σ(I), R_1_ = 0.0359, wR_2_ = 0.0784 for all pictures, GOOF = 0.990. The residual density peaks: Δρ = 0.184 e/A^3^ and −0.204 e/A^3^. Structure has been decoded and refined using the programs SHELXT-2014/5 [26] and SHELXL-2018/3 [27]. The X-ray diffraction data in the form of a CIF file has been deposited in the Cambridge Crystallographic Data Center (CCDC 2168324).

### Experimental Procedures

3,5-Dimethylisoxazole-4-sulfonyl chloride (**1**). To a cooled mixture of 33.8 mL of a chlorosulfonic acid and 4.06 mL of thionyl chloride, 5 mL of 3,5-dimethylisoxazole has been slowly added under stirring. A reaction mixture under stirring has been slowly heated to 120–130 °C for 4 h.

The reaction mixture has been cooled to room temperature and poured over 100 g of ice (be careful when adding it to ice; a reaction mixture can react vigorously with water).

The precipitated white residue has been filtered, washed with water and dissolved in 30 mL of chloroform. The solution has been washed with 40 mL of potassium carbonate 5% solution and dried over calcium chloride. Product (9) has been obtained as the white crystals. Its yield has been 2.28 g, m.p. 40–42 °C.

4-[(3,5-dimethyl-1,2-oxazol-4-yl)sulfonyl]cytisine (**2**). To a solution of 1.9 g (0.01 mol) of cytisine and 1.18 g (0.015 mol) of pyridine in 3 mL of dry acetonitrile, 1.95 g (0.01 mol) of sulfochloride 2 in 10 mL of dry acetonitrile has been added at room temperature. The color of a reaction mixture has been light yellow, and its residue has been yellow. A reaction mixture has been stirred for 1 h at 40 °C. Then, the reaction mixture has been cooled. A yellow residue has been filtered and washed with acetonitrile. Then a solvent has been distilled using a rotary evaporator. The residue was a thick yellow oil. Then 20 mL of a 5% solution of potassium carbonate has been added to the residue. After grinding, the thick oil has been reduced to a yellowish powder. The yield of product 2 has been 83%, m.p. 141–142 °C.

4-[(3-methyl-5-{(4-chlorophenyl)ethenyl}-1,2-oxazol-4-yl)sulfonyl]cytisine (**3**). To a solution of 0.91 g of sulfonamide 2 and 0.56 g of 4-chlorobenzaldehyde in 20 mL of ethanol, 2 mL of a 40% aqueous solution of potassium hydroxide has been added a room temperature. The color of the reaction mixture was cloudy, and then has become light yellow. The reaction mixture has been stirred and heated for 1 h at 60–70 °C. Then, the reaction mixture has been cooled to room temperature and added 15% hydrochloric acid solution to pH ≤ 3. A residue has been filtered and washed with water. The residue was a hygroscopic substance of a light yellow color, m.p. 217–219 °C.

## Figures and Tables

**Table 1:** Bond lengths (d, Å) in molecule **2**.

Bond	d	Bond	d
S(1)-O(3)	1.424(2)	C(9)-C(13)	1.523(4)
S(1)-O(2)	1.427(2)	C(9)-H(9A)	0.9800
S(1)-N(12)	1.634(2)	C(10)-H(10A)	0.9700
S(1)-C(4′)	1.749(3)	C(10)-H(10B)	0.9700
O(1)-C(2)	1.231(3)	C(11)-N(12)	1.472(4)
N(1)-C(6)	1.372(3)	C(11)-H(11A)	0.9700
N(1)-C(2)	1.400(3)	C(11)-H(11B)	0.9700
N(1)-C(10)	1.487(3)	N(12)-C(13)	1.484(3)
C(2)-C(3)	1.427(4)	C(13)-H(13A)	0.9700
C(3)-C(4)	1.348(5)	C(13)-H(13B)	0.9700
C(3)-H(3B)	0.9300	O(1′)-C(5′)	1.334(4)
C(4)-C(5)	1.396(4)	O(1′)-N(2′)	1.418(4)
C(4)-H(4A)	0.9300	N(2′)-C(3′)	1.304(4)
C(5)-C(6)	1.362(4)	C(3′)-C(4′)	1.412(4)
C(5)-H(5A)	0.9300	C(3′)-C(6′)	1.475(6)
C(6)-C(7)	1.500(4)	C(4′)-C(5′)	1.372(4)
C(7)-C(8)	1.526(4)	C(5′)-C(7′)	1.480(5)
C(7)-C(11)	1.532(4)	C(6′)-H(6′A)	0.9600
C(7)-H(7A)	0.9800	C(6′)-H(6′B)	0.9600
C(8)-C(9)	1.515(5)	C(6′)-H(6′C)	0.9600
C(8)-H(8A)	0.9700	C(7′)-H(7′A)	0.9600
C(8)-H(8B)	0.9700	C(7′)-H(7′B)	0.9600
C(9)-C(10)	1.512(4)	C(7′)-H(7′C)	0.9600

**Table 2:** Bond angles (ω, deg.) in molecule **2**.

Angle	ω	Angle	ω
O(3)-S(1)-O(2)	120.01(14)	N(1)-C(10)-H(10A)	108.6
O(3)-S(1)-N(12)	107.74(13)	C(9)-C(10)-H(10A)	108.6
O(2)-S(1)-N(12)	107.29(13)	N(1)-C(10)-H(10B)	108.6
O(3)-S(1)-C(4′)	106.65(14)	C(9)-C(10)-H(10B)	108.6
O(2)-S(1)-C(4′)	107.25(14)	H(10A)-C(10)-H(10B)	107.6
N(12)-S(1)-C(4′)	107.31(12)	N(12)-C(11)-C(7)	108.9(2)
C(6)-N(1)-C(2)	123.0(2)	N(12)-C(11)-H(11A)	109.9
C(6)-N(1)-C(10)	123.5(2)	C(7)-C(11)-H(11A)	109.9
C(2)-N(1)-C(10)	113.4(2)	N(12)-C(11)-H(11B)	109.9
O(1)-C(2)-N(1)	119.6(3)	C(7)-C(11)-H(11B)	109.9
O(1)-C(2)-C(3)	124.8(3)	H(11A)-C(11)-H(11B)	108.3
N(1)-C(2)-C(3)	115.6(3)	C(11)-N(12)-C(13)	113.4(2)
C(4)-C(3)-C(2)	121.2(3)	C(11)-N(12)-S(1)	117.54(17)
C(4)-C(3)-H(3B)	119.4	C(13)-N(12)-S(1)	115.02(19)
C(2)-C(3)-H(3B)	119.4	N(12)-C(13)-C(9)	109.5(2)
C(3)-C(4)-C(5)	120.8(3)	N(12)-C(13)-H(13A)	109.8
C(3)-C(4)-H(4A)	119.6	C(9)-C(13)-H(13A)	109.8
C(5)-C(4)-H(4A)	119.6	N(12)-C(13)-H(13B)	109.8
C(6)-C(5)-C(4)	120.1(3)	C(9)-C(13)-H(13B)	109.8
C(6)-C(5)-H(5A)	120.0	H(13A)-C(13)-H(13B)	108.2
C(4)-C(5)-H(5A)	120.0	C(5′)-O(1′)-N(2′)	108.7(2)
C(5)-C(6)-N(1)	119.3(2)	C(3′)-N(2′)-O(1′)	106.7(3)
C(5)-C(6)-C(7)	122.0(2)	N(2′)-C(3′)-C(4′)	110.1(3)
N(1)-C(6)-C(7)	118.7(2)	N(2′)-C(3′)-C(6′)	118.5(3)
C(6)-C(7)-C(8)	110.9(2)	C(4′)-C(3′)-C(6′)	131.4(3)
C(6)-C(7)-C(11)	111.1(2)	C(5′)-C(4′)-C(3′)	105.9(3)
C(8)-C(7)-C(11)	109.8(2)	C(5′)-C(4′)-S(1)	126.7(2)
C(6)-C(7)-H(7A)	108.3	C(3′)-C(4′)-S(1)	127.4(2)
C(8)-C(7)-H(7A)	108.3	O(1′)-C(5′)-C(4′)	108.6(3)
C(11)-C(7)-H(7A)	108.3	O(1′)-C(5′)-C(7′)	117.0(3)
C(9)-C(8)-C(7)	106.5(2)	C(4′)-C(5′)-C(7′)	134.4(3)
C(9)-C(8)-H(8A)	110.4	C(3′)-C(6′)-H(6′A)	109.5
C(7)-C(8)-H(8A)	110.4	C(3′)-C(6′)-H(6′B)	109.5
C(9)-C(8)-H(8B)	110.4	H(6′A)-C(6′)-H(6′B)	109.5
C(7)-C(8)-H(8B)	110.4	C(3′)-C(6′)-H(6′C)	109.5
H(8A)-C(8)-H(8B)	108.6	H(6′A)-C(6′)-H(6′C)	109.5
C(10)-C(9)-C(8)	111.1(2)	H(6′B)-C(6′)-H(6′C)	109.5
C(10)-C(9)-C(13)	112.2(2)	C(5′)-C(7′)-H(7′A)	109.5
C(8)-C(9)-C(13)	110.4(2)	C(5′)-C(7′)-H(7′B)	109.5
C(10)-C(9)-H(9A)	107.6	H(7′A)-C(7′)-H(7′B)	109.5
C(8)-C(9)-H(9A)	107.6	C(5′)-C(7′)-H(7′C)	109.5
C(13)-C(9)-H(9A)	107.6	H(7′A)-C(7′)-H(7′C)	109.5
N(1)-C(10)-C(9)	114.4(2)	H(7′B)-C(7′)-H(7′C)	109.5

**Table 3:** Torsion angles (τ, deg.) in molecule **2**.

Angle	Τ	Angle	τ
C(6)-N(1)-C(2)-O(1)	179.4(2)	O(3)-S(1)-N(12)-C(11)	168.7(2)
C(10)-N(1)-C(2)-O(1)	1.6(4)	O(2)-S(1)-N(12)-C(11)	38.1(2)
C(6)-N(1)-C(2)-C(3)	−0.9(4)	C(4′)-S(1)-N(12)-C(11)	−76.8(2)
C(10)-N(1)-C(2)-C(3)	−178.7(2)	O(3)-S(1)-N(12)-C(13)	−53.9(2)
O(1)-C(2)-C(3)-C(4)	−178.3(3)	O(2)-S(1)-N(12)-C(13)	175.6(2)
N(1)-C(2)-C(3)-C(4)	2.0(4)	C(4′)-S(1)-N(12)-C(13)	60.6(2)
C(2)-C(3)-C(4)-C(5)	−1.2(5)	C(11)-N(12)-C(13)-C(9)	−56.2(3)
C(3)-C(4)-C(5)-C(6)	−0.9(5)	S(1)-N(12)-C(13)-C(9)	164.5(2)
C(4)-C(5)-C(6)-N(1)	1.9(4)	C(10)-C(9)-C(13)-N(12)	−66.1(3)
C(4)-C(5)-C(6)-C(7)	−177.7(3)	C(8)-C(9)-C(13)-N(12)	58.4(3)
C(2)-N(1)-C(6)-C(5)	−1.1(4)	C(5′)-O(1′)-N(2′)-C(3′)	−0.2(4)
C(10)-N(1)-C(6)-C(5)	176.5(3)	O(1′)-N(2′)-C(3′)-C(4′)	0.4(4)
C(2)-N(1)-C(6)-C(7)	178.6(2)	O(1′)-N(2′)-C(3′)-C(6′)	178.5(4)
C(10)-N(1)-C(6)-C(7)	−3.8(3)	N(2′)-C(3′)-C(4′)-C(5′)	−0.4(4)
C(5)-C(6)-C(7)-C(8)	−148.2(3)	C(6′)-C(3′)-C(4′)-C(5′)	−178.2(4)
N(1)-C(6)-C(7)-C(8)	32.1(3)	N(2′)-C(3′)-C(4′)-S(1)	178.4(2)
C(5)-C(6)-C(7)-C(11)	89.3(3)	C(6′)-C(3′)-C(4′)-S(1)	0.6(6)
N(1)-C(6)-C(7)-C(11)	−90.3(3)	O(3)-S(1)-C(4′)-C(5′)	29.7(3)
C(6)-C(7)-C(8)-C(9)	−60.9(3)	O(2)-S(1)-C(4′)-C(5′)	159.4(3)
C(11)-C(7)-C(8)-C(9)	62.3(3)	N(12)-S(1)-C(4′)-C(5′)	−85.6(3)
C(7)-C(8)-C(9)-C(10)	63.5(3)	O(3)-S(1)-C(4′)-C(3′)	−148.9(3)
C(7)-C(8)-C(9)-C(13)	−61.6(3)	O(2)-S(1)-C(4′)-C(3′)	−19.1(3)
C(6)-N(1)-C(10)-C(9)	5.7(4)	N(12)-S(1)-C(4′)-C(3′)	95.9(3)
C(2)-N(1)-C(10)-C(9)	−176.5(2)	N(2′)-O(1′)-C(5′)-C(4′)	0.0(3)
C(8)-C(9)-C(10)-N(1)	−36.3(3)	N(2′)-O(1′)-C(5′)-C(7′)	−179.3(3)
C(13)-C(9)-C(10)-N(1)	87.8(3)	C(3′)-C(4′)-C(5′)-O(1′)	0.3(3)
C(6)-C(7)-C(11)-N(12)	63.2(3)	S(1)-C(4′)-C(5′)-O(1′)	−178.6(2)
C(8)-C(7)-C(11)-N(12)	−59.9(3)	C(3′)-C(4′)-C(5′)-C(7′)	179.3(4)
C(7)-C(11)-N(12)-C(13)	56.9(3)	S(1)-C(4′)-C(5′)-C(7′)	0.5(5)
C(7)-C(11)-N(12)-S(1)	−164.97(18)		

**Table 4:** The atomic coordinates (×10^4^) of structure in cell fraction **2**.

Atom	x	y	z
S(1)	1570(1)	6334(1)	2084(1)
O(1)	3784(4)	4085(3)	6024(2)
O(2)	−479(3)	5822(3)	1848(2)
O(3)	2015(4)	7585(3)	2713(2)
N(1)	3553(3)	3144(2)	4497(2)
C(2)	2863(4)	3270(3)	5396(2)
C(3)	1088(5)	2419(4)	5507(2)
C(4)	195(5)	1522(4)	4792(3)
C(5)	965(4)	1421(4)	3914(2)
C(6)	2621(4)	2250(3)	3765(2)
C(7)	3456(4)	2222(3)	2821(2)
C(8)	5760(4)	2453(4)	2978(2)
C(9)	6137(4)	3981(4)	3445(2)
C(10)	5433(4)	4025(4)	4428(2)
C(11)	2442(4)	3421(3)	2127(2)
N(12)	2927(3)	4914(3)	2551(2)
C(13)	5147(4)	5205(4)	2775(2)
O(1′)	4206(5)	7819(3)	−55(2)
N(2′)	2656(6)	6928(3)	−579(2)
C(3′)	1633(5)	6326(4)	55(2)
C(4′)	2446(4)	6796(3)	997(2)
C(5′)	4050(5)	7722(3)	885(2)
C(6′)	−125(9)	5360(5)	−294(3)
C(7′)	5530(5)	8596(4)	1557(3)

**Table 5:** Effect of compound **2** on blood viscosity (mPa × s) at various spindle speeds in the in vitro model of blood hyperviscosity.

Tested Parameter	Blood Viscosity (mPa × s) at Various Spindle Speeds, rpm							
	2	4	6	8	12	20	40	60
Reference viscosity, *n* = 2	2.71 ± 0.05	2.25 ± 0.02	2.05 ± 0.01	1.80 ± 0.04	1.68 ± 0.04	1.45 ± 0.02	1.30 ± 0.6	1.27 ± 0.05
Blood viscosity after 1 h of incubation at 43 °C in a control, *n* = 4	8.75 ± 0.26	7.05 ± 0.14	5.30 ± 0.07	4.46 ± 0.10	3.65 ± 0.29	3.33 ± 0.42	3.01 ± 0.37	2.85 ± 0.41
	*p*_1_ = 0.0001	*p*_1_ = 0.00002	*p*_1_ = 0.00001	*p*_1_ = 0.0001	*p*_1_ = 0.0113	*p*_1_ = 0.0420	*p*_1_ = 0.0368	*p*_1_ = 0.0627
Blood viscosity after 1 h of incubation at 43 °C, samples with compound 2, *n* = 4	6.83 ± 0.10	5.21 ± 0.06	4.74 ± 0.08	3.81 ± 0.08	3.01 ± 0.6	2.67 ± 0.9	2.32 ± 0.15	2.17 ± 0.10
	*p*_1_ = 0.00001 *p*_2_ = 0.0005	*p*_1_ = 0.00001 *p*_2_ = 0.00002	*p*_1_ = 0.00003 *p*_2_ = 0.0021	*p*_1_ = 0.0001 *p*_2_ = 0.0018	*p*_1_ = 0.0261 *p*_2_ = 0.1511	*p*_1_ = 0.0118 *p*_2_ = 0.2044	*p*_1_ = 0.0109 *p*_2_ = 0.1332	*p*_1_ = 0.0040 *p*_2_ = 0.1580

Note: *n*—number of samples in a group; *p*—significance level; *p*_1_ < 0.05—statistically significant differences compared to original values; *p*_2_ < 0.05—statistically significant differences compared to proper values in the control samples.

**Table 6:** Effect of pentoxifiline on blood viscosity (mPa × s) at various spindle speeds in the in vitro blood hyperviscosity model.

Tested Parameter	Blood Viscosity (mPa × s) at Various Spindle Speeds, rpm							
	2	4	6	8	12	20	40	60
Reference viscosity, *n* = 2	5.94 ± 0.59	4.90 ± 0.43	4.10 ± 0.38	3.87 ± 0.34	3.40 ± 0.29	2.69 ± 0.26	2.32 ± 0.12	2.21 ± 0.12
Blood viscosity after 1 h of incubation at 43 °C in a control, *n* = 4	7.53 ± 0.45	6.36 ± 0.40	5.79 ± 0.44	5.19 ± 0.31	4.37 ± 0.13	3.56 ± 0.15	2.76 ± 0.09	2.53 ± 0.07
	*p*_1_ = 0.0519	*p*_1_ = 0.0364	*p*_1_ = 0.0250	*p*_1_ = 0.0184	*p*_1_ = 0.0026	*p*_1_ = 0.0065	*p*_1_ = 0.0098	*p*_1_ = 0.0218
Blood viscosity after 1 h of incubation at 43 °C, samples with pentoxifylline, *n* = 4	7.03 ± 0.43	5.81 ± 0.30	5.00 ± 0.21	4.56 ± 0.16	4.05 ± 0.10	3.24 ± 0.14	2.56 ± 0.08	2.39 ± 0.07
	*p*_1_ = 0.1584 *p*_2_ = 0.4306	*p*_1_ = 0.1009 *p*_2_ = 0.2800	*p*_1_ = 0.0357 *p*_2_ = 0.1205	*p*_1_ = 0.0532 *p*_2_ = 0.0855	*p*_1_ = 0.0171 *p*_2_ = 0.0631	*p*_1_ = 0.0563 *p*_2_ = 0.1353	*p*_1_ = 0.0960 *p*_2_ = 0.0999	*p*_1_ = 0.1887 *p*_2_ = 0.1590

Note: *n*—number of samples in a group; *p*—significance level; *p*_1_ < 0.05—statistically significant differences compared to original values; *p*_2_ < 0.05—statistically significant differences compared to proper values in the control samples.

## Data Availability

Not applicable.

## Supplementary Materials

The following supporting information can be downloaded at: https://www.mdpi.com/article/10.3390/plants12010137/s1. Pages 1–3, Experimental Procedures, Spectroscopic and physical data; Figure S1: ^1^H and ^13^C NMR spectra of compound **2**; Figures S2–S5: spectra and structural correlations in COSY and HMQC spectra of compound **2**; Figure S6: ^1^H and ^13^C NMR spectra of compound **3**; Figures S7–S10: spectra and structural correlations in COSY and HMQC spectra of compound **3**; Figures S11–S12: Mass spectra of compounds **2**, **3**; Tables S1–S3: X-ray data of compound **2**. The cif-file is presented singly.
