# Supplementary material for: Synthesis, Properties and Spatial Structure of 4-[(3,5-Dimethyl-1,2-oxazol-4-yl)sulfonyl]cytisine
Source: Plants (Basel). 2022 Dec 27;12(1):137. doi: 10.3390/plants12010137 (PMC9823373; doi:10.3390/plants12010137)
Supplement: Supplementary file 1 [file plants-12-00137-s001.zip › plants-2020412-supplementary.pdf]

Supporting information

Synthesis, Properties and Spatial Structure of 4-[(3,5-Dimethyl-1,2-oxazol-4-yl)sulfonyl]cytisine

Marat K. Ibrayev [1,2,*], Oralgazy A. Nurkenov [1,3], Zhanara B. Rakhimberlinova [1], Altynaray T. Takibayeva [1], Dastan M. Turdybekov [1], Tulegen M. Seilkhanov [4], Meruyert B. Issabayeva [5,*], Assel A. Kelmyalene [1], Assel T. Kezdikbayeva [2] and Anel Z. Mendibayeva [3]

[1] Abylkas Saginov Karaganda Technical University, Ave. N. Nazarbayev, 56, Karaganda 100027, Kazakhstan
[2] Faculty of Chemistry, Karaganda Buketov University, st. University 28, Karaganda 100024, Kazakhstan
[3] Institute of Organic Synthesis and Coal Chemistry of Republic of Kazakhstan, st. Alikhanov, 1, Karaganda 100008, Kazakhstan
[4] Laboratory of Engineering Profile NMR Spectroscopy, Sh. Ualikhanov Kokshetau University, Kokshetau 020008, Kazakhstan
[5] School of Pharmacy, Medical University of Karaganda, Karaganda 100008, Kazakhstan
* Correspondence: mkibr@mail.ru (M.K.I.); missabaeva@mail.ru (M.B.I.);
Tel.: +7-700-3106646 (M.K.I.)

Table of contents

Experimental 1
General Information 1
Experimental Procedures 2
Spectroscopic and physical data 3
Copies of NMR Spectra of Products 4
Copies of MS Spectra of Products 12
X-Ray Structural Study of Product 13

Experimental

General Information

FTIR spectra were obtained with an Agilent Cary 630 spectrophotometer in a thin sample layer on a crystal attachment. ^{1}H and ^{13}C NMR spectra were recorded on JNM-ECA Jeol 400 spectrometer (fre-quency 399.78 and 100.53 MHz, respectively) with using of CDCl3 solvent. The chemical shifts have been measured relative to signals of residual protons or carbon atoms of deuterated chloroform.

Chromato-mass spectrometric studies were carried out on a Trace GC Ultra chromatograph with a DSQ II mass-selective detector in the electron ionization mode (70 eV) on a Thermo TR-5 MS quartz capillary column, 15 m long, 0.25 mm inner diameter, with a film thickness of the stationary phase of 0.25 μm. Splitless input mode was used. Carrier gas discharge 20 ml/min. The velocity of the carrier gas (helium) is 1 ml/min. Evaporator temperature 200°C, transition chamber temperature 200°C, ion source temperature 200°C. The temperature of the column thermostat was changed according to the program: from 15 (5 min delay) to 220°C at a rate of 20°C per minute, to 290° at a rate of 15° per minute. The total analysis time was 30 min. The volume of the injected sample is 1 μl. Chromatograms were recorded in TIC mode. The range of mass scanning is 30 - 450 amu.

Melting points were determined using a Stuart SMP10 hot bench. Monitoring of the reaction course and the purity of the products was carried out by TLC on Sorbfil plates and visualized using iodine vapor or UV light.

Experimental Procedures

3,5-Dimethylisoxazole-4-sulfonyl chloride (1). To a cooled mixture of 33.8 mL of a chlorosulfonic acid and 4.06 mL of thionyl chloride, 5 mL of 3,5-dimethylisoxazole has been slowly added under stirring. A reaction mixture under stirring has been slowly heated to 120-130°C for 4 h.

The reaction mixture has been cooled to a room temperature and poured over 100 g of ice (be careful when added to ice, a reaction mixture can react vigorously with water).

The precipitated white residue has been filtered, washed with water and dissolved in 30 ml of chloroform. Solution has been washed with 40 ml of potassium carbonate 5% solution and dried over calcium chloride. Product (9) has been obtained as the white crystals. Its yield has been 2.28 g, m.p. 40-2°C.

4-[(3,5-dimethyl-1,2-oxazol-4-yl)sulfonyl]cytisine (2). To a solution of 1.9 g (0.01 mol) of cytisine and 1.18 g (0.015 mol) of pyridine in 3 ml of dry acetonitrile, 1.95 g (0.01 mol) of sulfochloride 2 in 10 ml of dry acetonitrile has been added at a room temperature. A color of a reaction mixture has been light yellow, and its residue has been yellow. A reaction mixture has been stirred for 1 h at 40°C. Then a reaction mixture has been cooled. A yellow residue has been filtered and washed with acetonitrile. Then a solvent has been distilled with using a rotary evaporator. A residue has been thick yellow oil. Then 20 ml of a 5% solution of potassium carbonate has been added to a residue. After grinding, the thick oil has been a yellowish powder. The yield of product 2 has been 83%, m.p. 141-142°C.

4-[(3-methyl-5-{(4-chlorophenyl)ethenyl}-1,2-oxazol-4-yl)sulfonyl]cytisine (3). To a solution of 0.91 g of sulfonamide 2 and 0.56 g of 4-chlorobenzaldehyde in 20 ml of ethanol, 2 ml of a 40% aqueous solution of potassium hydroxide has been added at a room temperature. A color of a reaction mixture has been cloudy, and then has become light yellow. A reaction mixture has been stirred and heated for 1 h at 60-70°C. Then, a reaction mixture has been cooled to a room temperature and added 15% hydrochloric acid solution to pH≤3. A residue has been filtered and washed with water. The residue was a hygroscopic substance, light yellow color, m.p. 217-219°C.

Spectroscopic and physical data

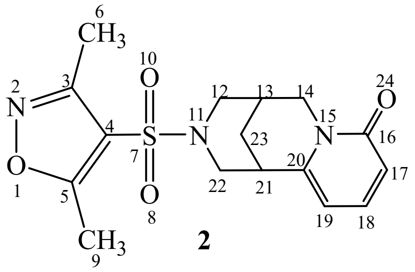<br>Chemical Formula: $C_{16}H_{19}N_3O_4S$<br>Molecular Weight: 349,41	**4-[(3,5-dimethyl-1,2-oxazol-4-yl)sulfonyl]-cytisine (2).**<br>Yield: 83%; yellowish powder, mp. 141-142 °C (2-PrOH).<br>^{1}H NMR (399.78 MHz, DMSO-d$_6$, δ, ppm, *J*/ Hz): 1.76-1.85 m (2H, H-23ax,23eq), 2.49 s (4H, H-9,9,9,13), 2.83 d (1H, H-12ax, 2J 11.4 Hz), 2.92 d (1H, H-12eq, 2J 11.4 Hz), 3.22 s (1H, H-21), 3.56 d (1H, H-22ax, 2J 10.8 Hz), 3.61 d (1H, H-22eq, 2J 10.8 Hz), 3.73-3.83 m (2H, H-14ax,14eq) ppm. 6.30 br. s (1H, H-17), 6.36 d (1H, H-19, 3J 9.2 Hz), 7.43 t (1H, 2J 6.6 Hz) ppm. H-6,6,6 2.12 ppm, H-9,9,9 2.49 ppm.<br>^{13}C NMR (100.53 MHz, DMSO-d$_6$) δ ppm 24.07 (C-23), 26.59 (C-13), 33.60 (C-21), 49.70 (C-14), 51.20 (C-22), 52.61 (C-12), 106.91 (C-19), 115.90 (C-17), 140.37 (C-18), 150.60 (C-20) и 162.45 (C-16) м.д.. 11.04 (C-6), 12.95 (C-9), 113.30 (C-4), 158.21 (C-3) и 174.45 (C-5).
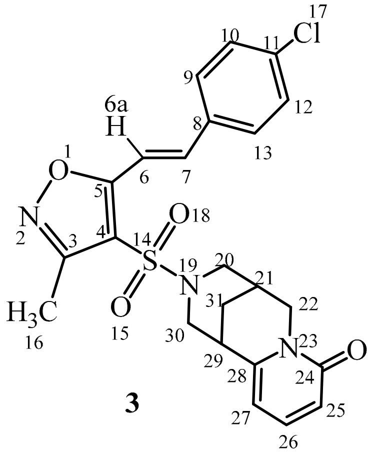<br>Chemical Formula: $C_{23}H_{22}ClN_3O_4S$<br>Molecular Weight: 471,96	**4-[(3-methyl-5-{(4-chlorophenyl)ethenyl}-1,2-oxazol-4-yl)sulfonyl]cytisine (3).** Yield: 76%; Hygroscopic substance, light yellow color, mp 217-219 °C (2-PrOH).<br>^{1}H NMR (399.78 MHz, DMSO-d$_6$, δ, ppm, *J*/ 1.74-1.77 m (2H, H-31ax,31eq), 2.45 s (1H, H-21), 2.81 d (1H, H-20ax, 2J 11.6 Hz), 2.91 d (1H, H-20eq, 2J 11.6 Hz), 3.17 s (1H, H-29), 3.29-3.83 m (H-22ax,22eq,30ax,30eq) ppm. 6.15-6.17 m (1H, H-27), 6.22-6.24 m (1H, H-25), 7.30-7.33 m (1H, H-26) ppm. H-16,16,16 - 2.50 s, H-10,12 - 7.46-7.49, H-9,13 - 7.69-7.71 ppm.<br>^{13}C NMR (100.53 MHz, DMSO-d6) δ ppm 24.12 (C-31), 26.75 (C-21), 33.87 (C-29), 49.27 (C-30), 51.07 (C-22), 52.80 (C-20), 105.65 (C-27), 117.13 (C-25), 140.08 (C-26), 150.11 (C-28) и 162.62 (C-24), 13.01 (C-16), 112.57 (C-4), 129.58 (C-6), 130.28 и 131.64 (C-9,10,12,13), 133.96 (C-11), 138.52 (C-7,8), 150.11 (C-3), 158.52 (C-5).

Copies of NMR Spectra of Products

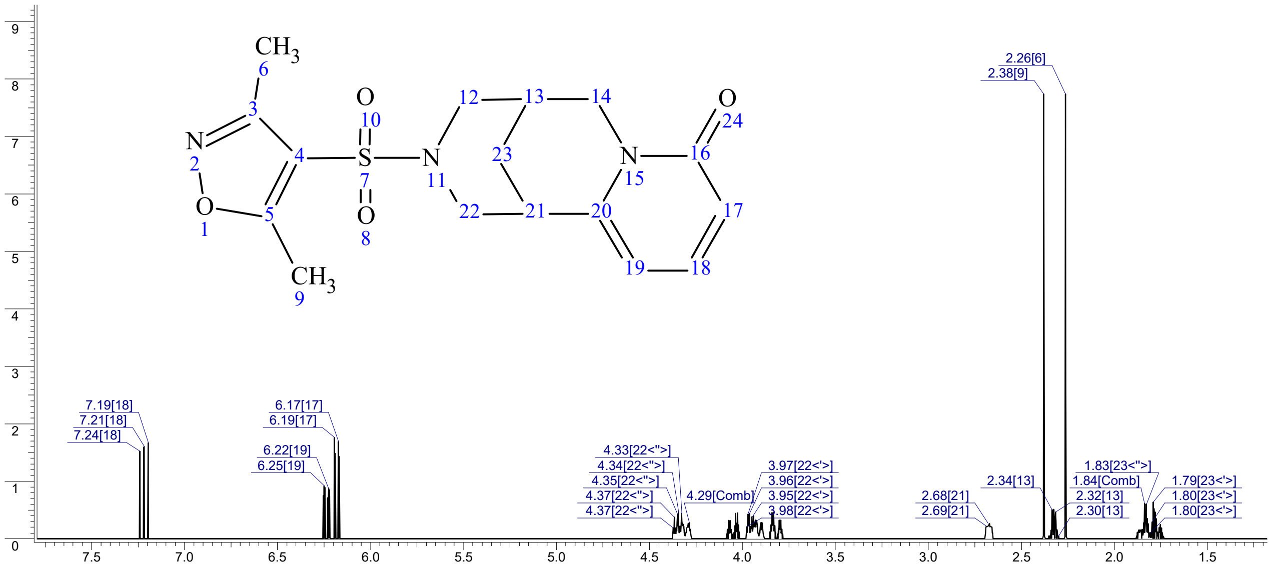

Group	nH	Shift	Error
6	3	2.26	0.18
9	3	2.38	0.30
12<">	1	4.30	0.45
12<'>	1	3.91	0.44
13	1	2.33	0.26
14<">	1	4.05	0.07
14<'>	1	3.82	0.04
17	1	6.18	0.16

Group	nH	Shift	Error
18	1	7.22	0.11
19	1	6.24	0.22
21	1	2.67	0.20
22<">	1	4.35	0.45
22<'>	1	3.95	0.44
23<">	1	1.84	0.44
23<'>	1	1.77	0.44

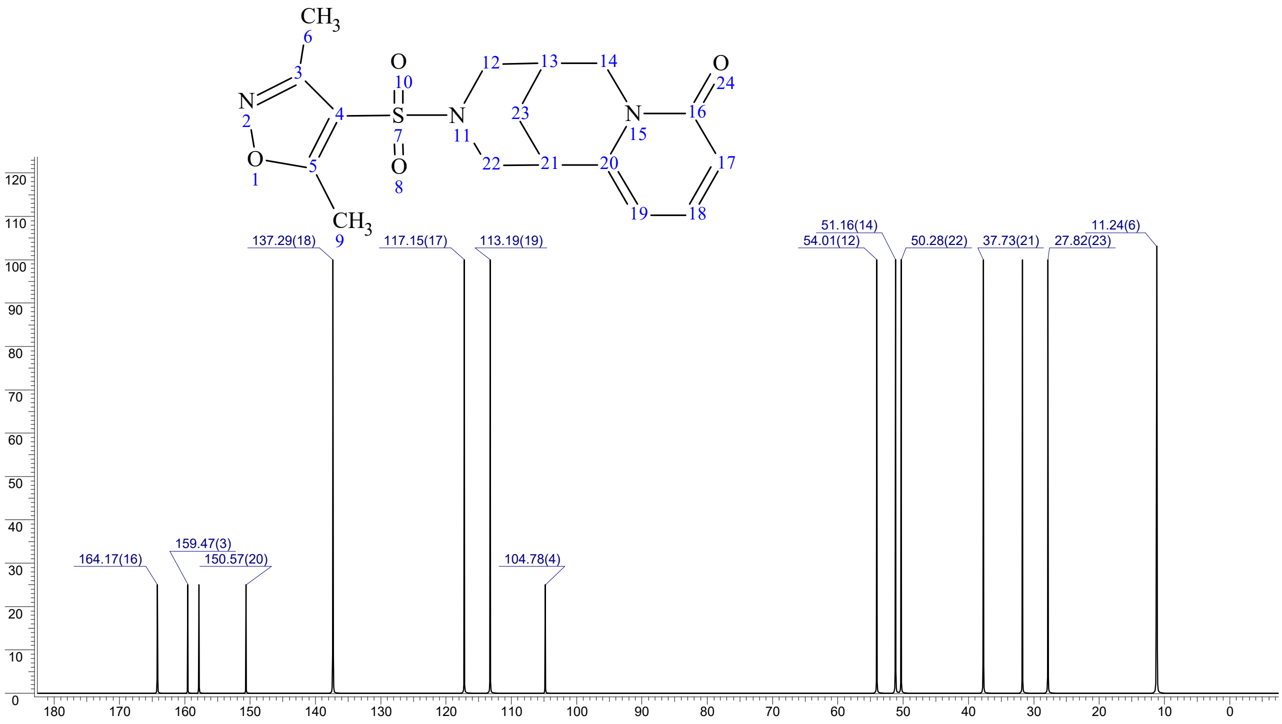

Carbon No.	CHn	Chem. Shifts	Conf. Limits
3	C	159.47	6.5
4	C	104.78	13.2
5	C	157.8	6.5
6	CH_3	11.24	2.9
9	CH_3	11.17	2.1
12	CH_2	54.01	1.7
13	CH	31.72	3.2
14	CH_2	51.16	1.1

Carbon No.	CHn	Chem. Shifts	Conf. Limits
16	C	164.17	2.5
17	CH	117.15	2.7
18	CH	137.29	1.4
19	CH	113.19	1.7
20	C	150.57	1
21	CH	37.73	4.4
22	CH_2	50.28	5.8
23	CH_2	27.82	5.3

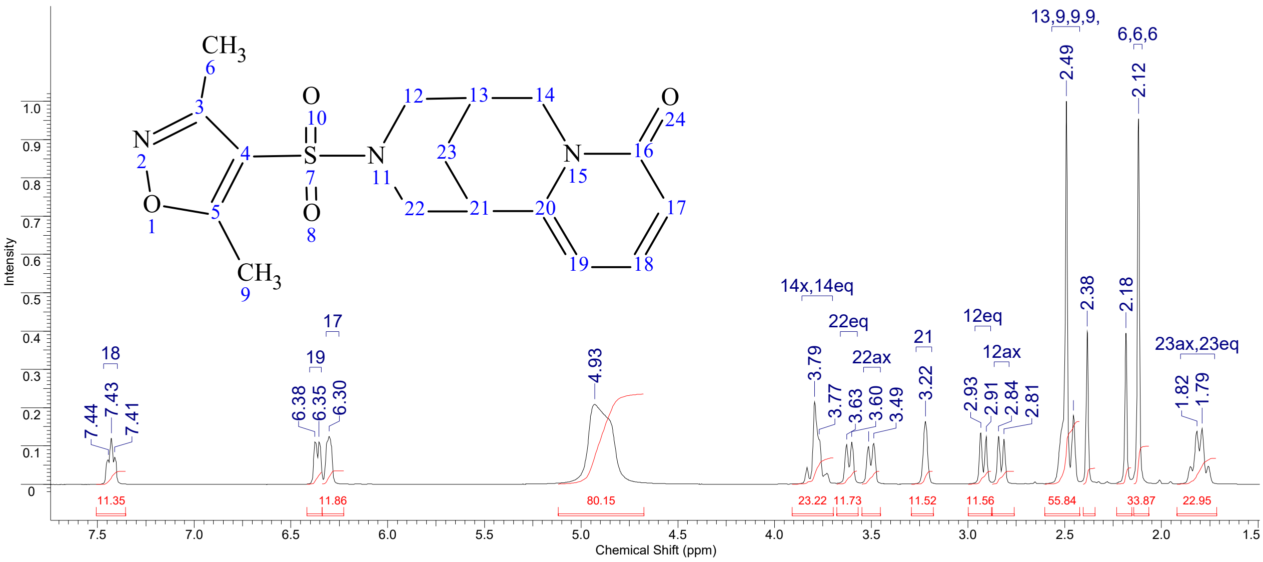

No.	(ppm)	(Hz)	Height
1	1.79	715.7	0.1453
2	1.82	726.3	0.1379
3	2.12	847.2	0.9546
4	2.18	872.8	0.3948
5	2.38	953.0	0.3988
6	2.45	981.4	0.1787
7	2.49	996.0	1.0000
8	2.81	1124.7	0.1171
9	2.84	1136.1	0.1251
10	2.91	1161.8	0.1235
11	2.93	1172.8	0.1339
12	3.22	1287.3	0.1637
13	3.49	1394.4	0.1066

No.	(ppm)	(Hz)	Height
14	3.51	1405.0	0.0976
15	3.60	1439.3	0.1106
16	3.63	1450.3	0.1028
17	3.77	1506.6	0.1168
18	3.79	1516.2	0.2145
19	4.93	1970.5	0.2085
20	6.30	2519.6	0.1246
21	6.35	2540.2	0.1100
22	6.38	2549.4	0.1107
23	7.41	2962.9	0.0703
24	7.43	2969.8	0.1198
25	7.44	2976.2	0.0634

No.	Annotation	(ppm)
1	23ax,23eq	[1.72 .. 1.90]
2	6,6,6	[2.10 .. 2.14]
3	13,9,9,9,	[2.42 .. 2.57]
4	12ax	[2.79 .. 2.86]
5	12eq	[2.88 .. 2.96]
6	21	[3.19 .. 3.27]
7	22ax	[3.45 .. 3.54]
8	22eq	[3.57 .. 3.66]
9	14x,14eq	[3.70 .. 3.86]
10	17	[6.25 .. 6.32]
11	19	[6.34 .. 6.40]
12	18	[7.40 .. 7.47]

No.	(ppm)	Value	Absolute Value
1	[6.34 .. 6.42]	11.168	1.09728e+1
2	[7.36 .. 7.51]	11.354	1.11557e+1
3	[3.45 .. 3.55]	11.492	1.12911e+1
4	[3.18 .. 3.29]	11.517	1.13161e+1
5	[2.76 .. 2.87]	11.548	1.13464e+1
6	[2.88 .. 3.00]	11.563	1.13607e+1
7	[3.57 .. 3.68]	11.731	1.15266e+1
8	[6.23 .. 6.34]	11.861	1.16539e+1

No.	(ppm)	Value	Absolute Value
9	[2.34 .. 2.40]	14.147	1.39000e+1
10	[2.15 .. 2.23]	14.747	1.44893e+1
11	[1.71 .. 1.92]	22.950	2.25498e+1
12	[3.70 .. 3.91]	23.218	2.28124e+1
13	[2.07 .. 2.14]	33.871	3.32797e+1
14	[2.42 .. 2.60]	55.839	5.48642e+1
15	[4.67 .. 5.12]	80.147	7.87480e+1

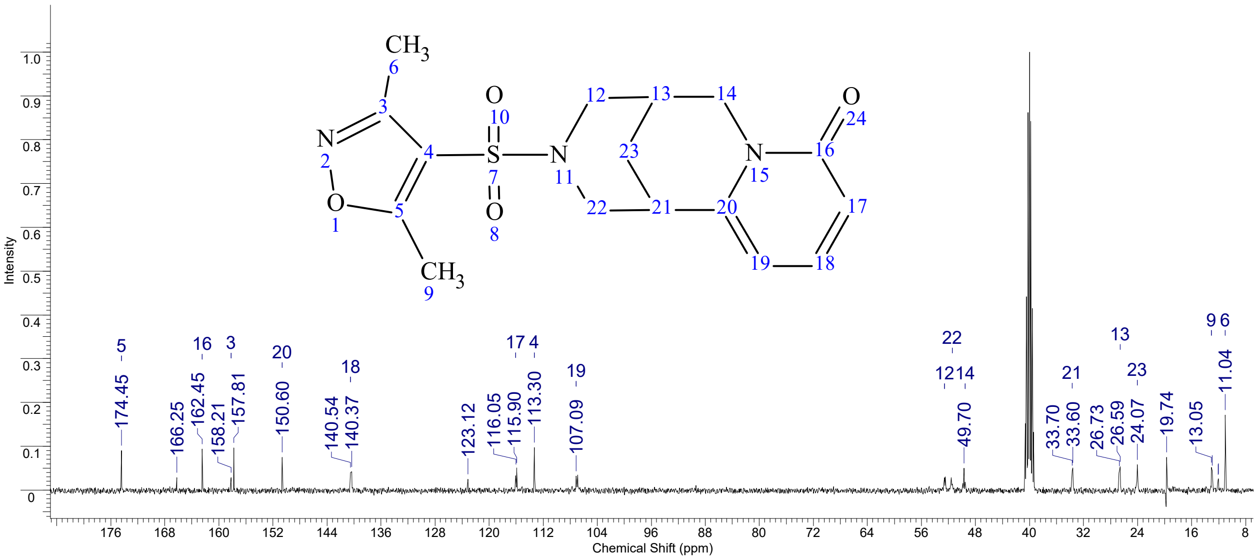

No.	(ppm)	(Hz)	Height
1	11.04	1110.0	0.1713
2	12.02	1208.8	0.0255
3	12.11	1217.4	0.0234
4	12.95	1301.7	0.0442
5	13.05	1312.3	0.0518
6	19.74	1984.2	0.0748
7	24.07	2419.3	0.0577
8	26.59	2673.3	0.0535
9	26.73	2686.7	0.0435
10	33.60	3377.8	0.0509
11	33.70	3387.4	0.0441
12	49.70	4995.7	0.0499
13	107.09	10765.6	0.0320

No.	(ppm)	(Hz)	Height
14	113.30	11389.6	0.0970
15	115.90	11651.3	0.0510
16	116.05	11665.6	0.0474
17	123.12	12376.8	0.0251
18	140.37	14110.7	0.0415
19	140.54	14127.9	0.0396
20	150.60	15139.1	0.0752
21	157.81	15863.7	0.0959
22	158.21	15904.0	0.0287
23	162.45	16330.5	0.0934
24	166.25	16712.0	0.0280
25	174.45	17536.2	0.0897

No.	Annotation	(ppm)
1	6	11.04
2	9	13.05
3	23	24.07
4	13	26.59
5	21	33.70
6	14	49.53
7	22	51.45
8	12	52.61

No.	Annotation	(ppm)
9	19	107.09
10	4	113.30
11	17	116.07
12	18	140.54
13	20	150.60
14	3	158.21
15	16	162.45
16	5	174.45

Figure S1. ^{1}H (399.78 MHz, DMSO-d6) and ^{13}C (100.53 MHz, DMSO-d6) NMR Spectra of **2**

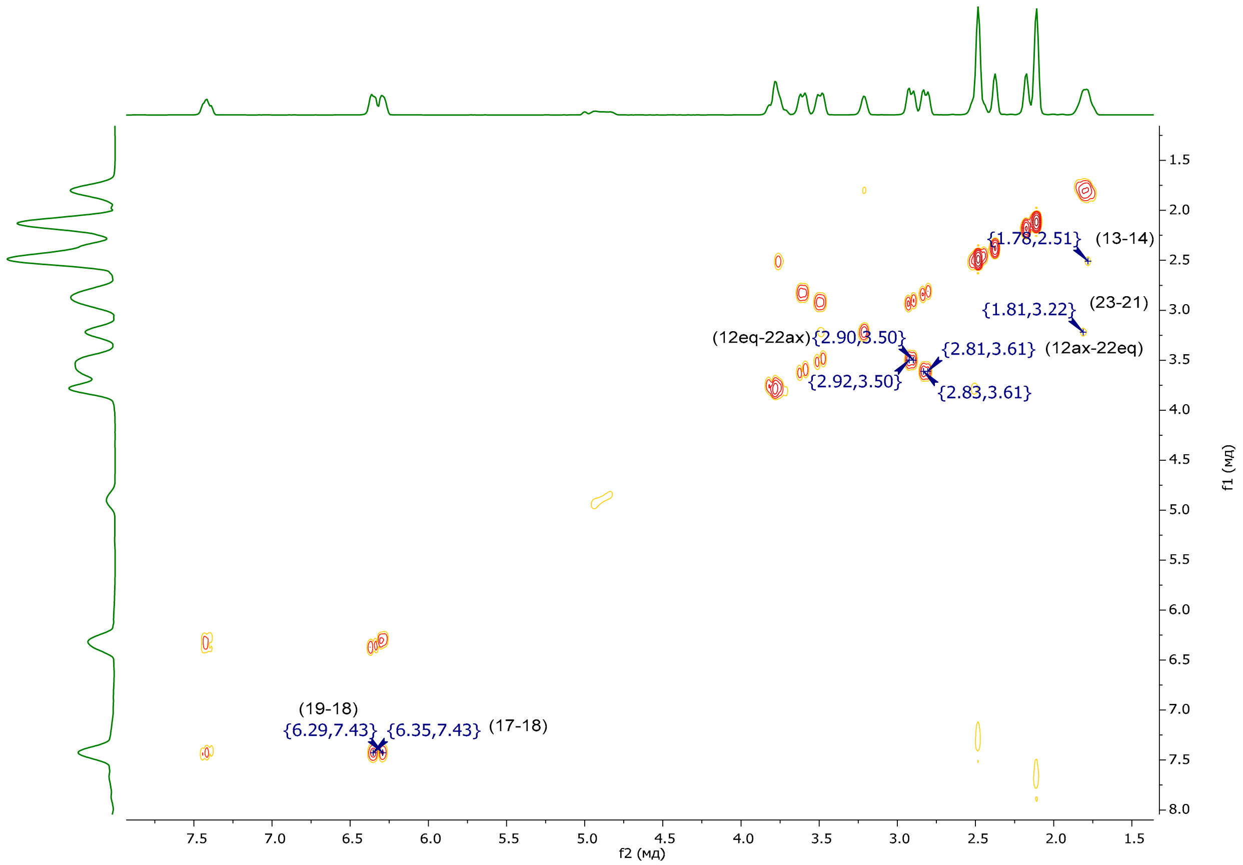

Figure S2. Cosy of **2** in_DMSO

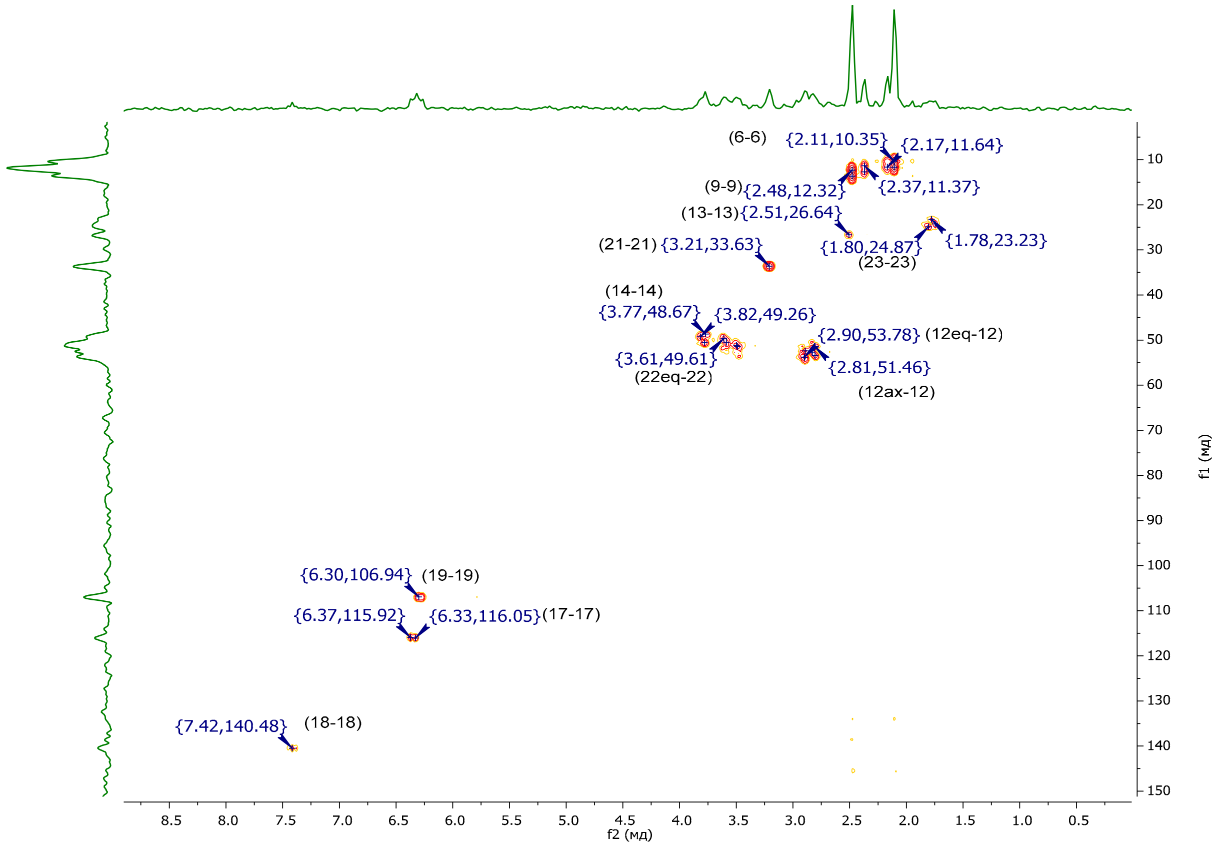

Figure S3 Hmqc of **2** in_DMSO

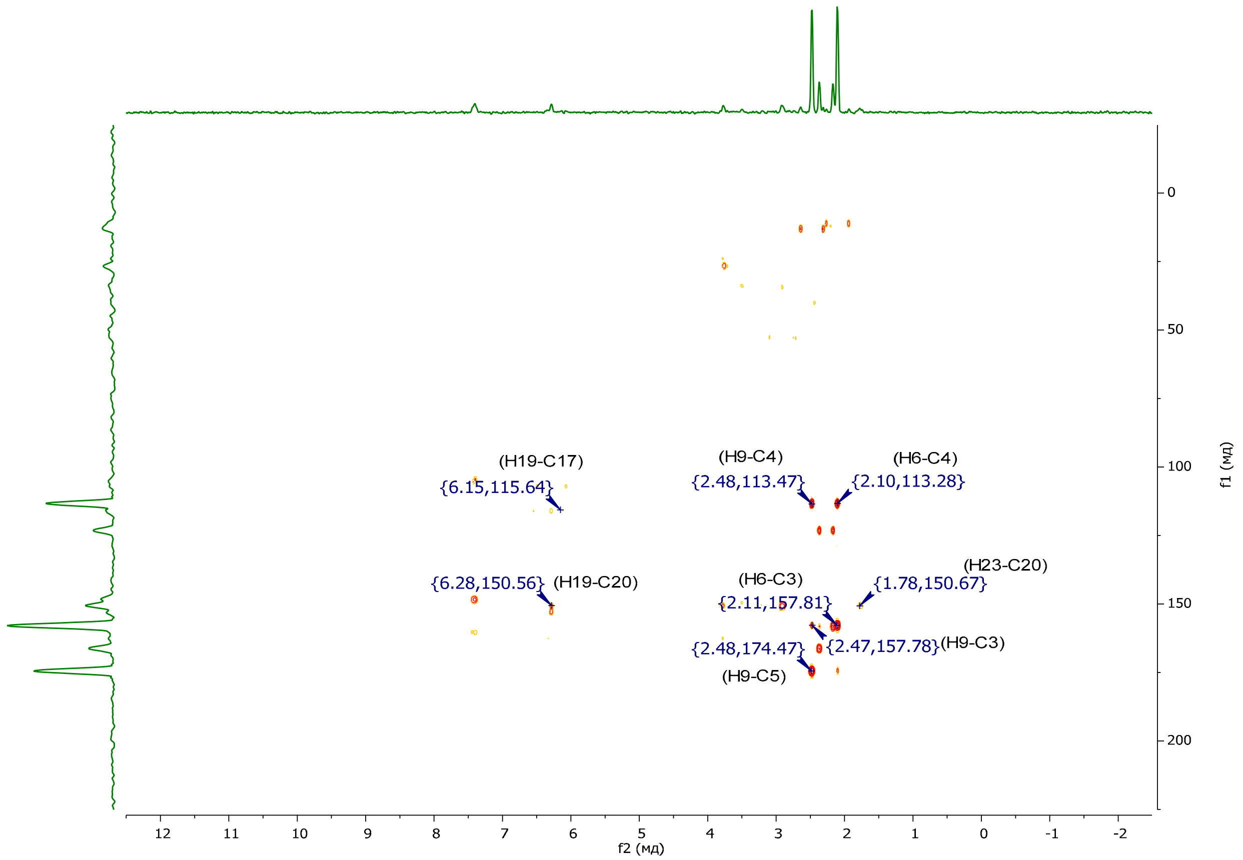

Figure S4. Hmbc of **2** in_DMSO

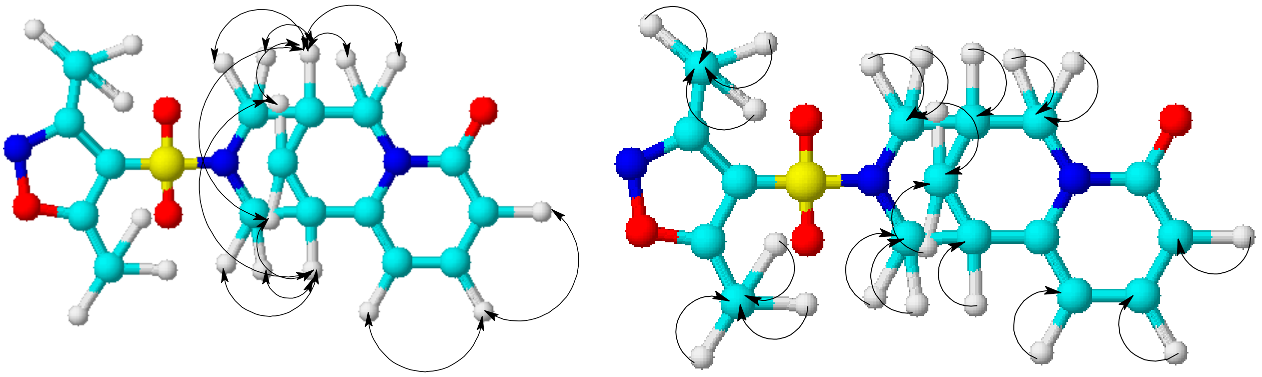

a b

Figure S5. The structural correlations in COSY (a) and HMQC (b) spectra of compound **2**

The 1H-1H COSY spectra of compound 2 have demonstrated the spin-spin correla-tions through three proton bonds of near methylene-methylene, methine-methylene and methine-methine groups of H23-H21 (1.80, 3.21 and 3.21, 1.80), H13-H14 (2.50, 3.76 and 3.76, 2.50), H12ax-H22eq (2.81, 3.60 and 3.60, 2.81), H12eq-H22ax (2.90, 3.49 and 3.49, 3.49), H19-H18 (6.28, 7.42 and 7.42. 6.28) and H17-H18 (6.31, 7.41 and 7.41, 6.31) ppm.

Hetero-nuclear couplings of protons with carbon atoms through a single bond have been established by 1H-13C HMQC spectroscopy for the following pairs in a compound: H6-C6 (2.10, 11.37), H9-C9 (2.47, 12.85), H23-C23 (1.79, 24.74), H13-C13 (2.50, 26.87), H21-C21 (3.20, 33.83), H12ax-C12 (2.81, 52.89), H12eq-C12 (2.93, 52.44), H22ax-C22 (3.46, 51.14),H22eq-C22 (3.60, 51.38), H14-C14 (3.76, 50.01), H19-C19 (6.28, 107.35), H17-C17 (6.34, 116.27) and H18-C18 (7.42, 140.69) ppm.

Hetero-nuclear couplings of protons with carbon atoms through two or more bonds have been determined by 1H-13C HMBC spectroscopy for the following pairs in a compound: H19-C17 (6.28, 116.38), H19-C20 (6.28, 150.54); H9-C4 (2.47, 114.10), H9-C3 (2.47, 157.09), H9-C5 (2.47, 175.02); H6-C4 (2.10, 114.11), H6-C3 (2.10, 158.22) and H23-C20 (1.78, 150.54) ppm.

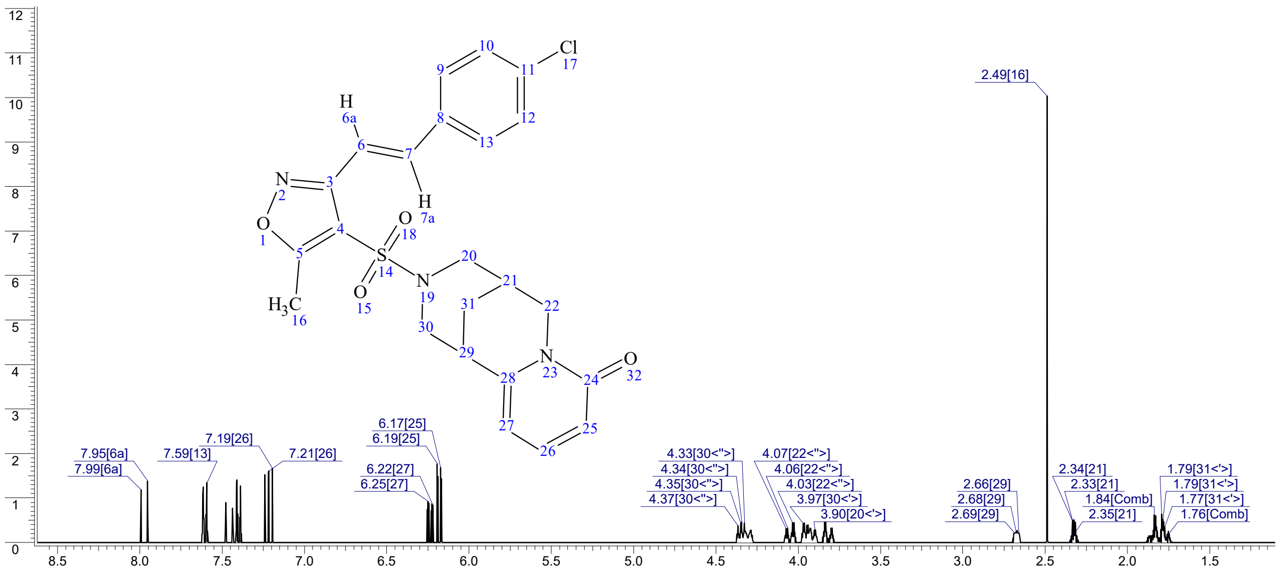

Group	nH	Shift	Error	Group	nH	Shift	Error
6a	1	7.97	0.38	22<">	1	4.05	0.07
7a	1	7.46	0.27	22<'>	1	3.82	0.04
9	1	7.60	0.07	25	1	6.18	0.16
10	1	7.40	0.05	26	1	7.22	0.11
12	1	7.40	0.05	27	1	6.24	0.22
13	1	7.60	0.07	29	1	2.67	0.20
16	3	2.49	0.29	30<">	1	4.35	0.45
20<">	1	4.30	0.45	30<'>	1	3.95	0.44
20<'>	1	3.91	0.44	31<">	1	1.84	0.44
21	1	2.33	0.26	31<'>	1	1.77	0.44

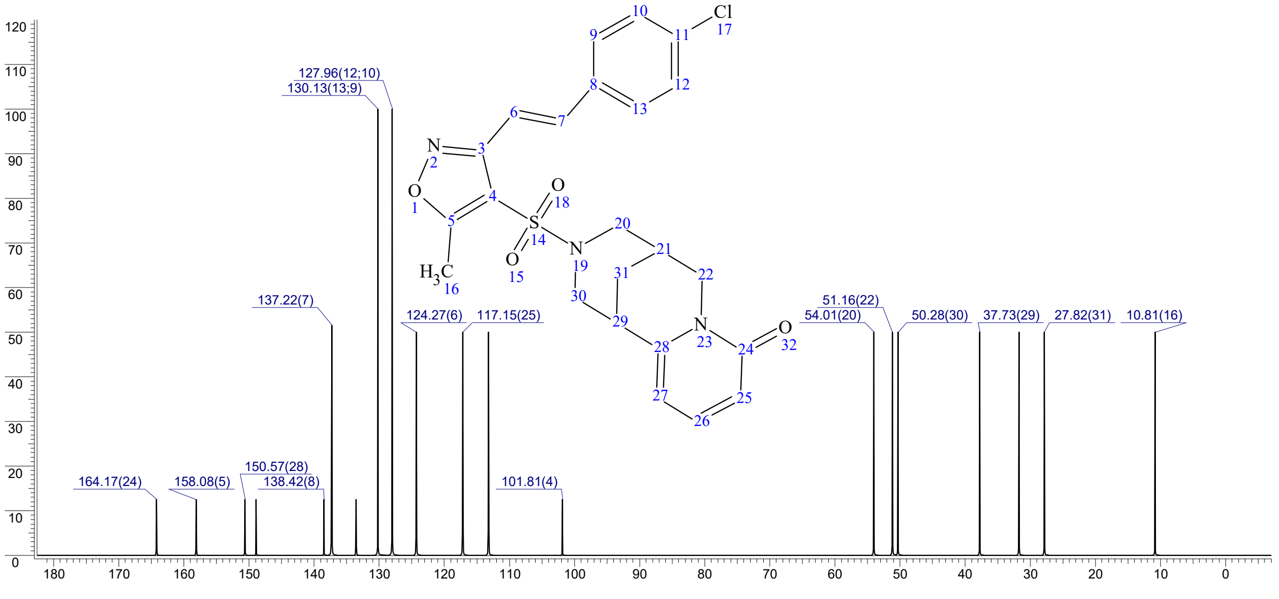

Carbon No.	CHn	Chem. Shifts	Conf. Limits	Carbon No.	CHn	Chem. Shifts	Conf. Limits
3	C	148.83	6.7	20	CH_2	54.01	1.7
4	C	101.81	13.2	21	CH	31.72	3.2
5	C	158.08	6.5	22	CH_2	51.16	1.1
6	CH	124.27	5.1	24	C	164.17	2.5
7	CH	137.22	3.1	25	CH	117.15	2.7
8	C	138.42	1.1	26	CH	137.29	1.4
9	CH	130.13	0.7	27	CH	113.19	1.7
10	CH	127.96	1	28	C	150.57	1
11	C	133.54	1.3	29	CH	37.73	4.4
12	CH	127.96	1	30	CH_2	50.28	5.8
13	CH	130.13	0.7	31	CH_2	27.82	5.3
16	CH_3	10.81	2				

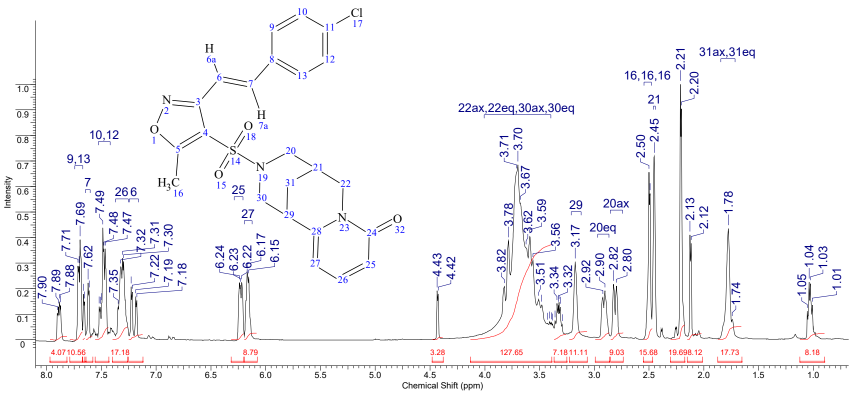

No.	(ppm)	(Hz)	Height	No.	(ppm)	(Hz)	Height	No.	(ppm)	(Hz)	Height	No.	(ppm)	(Hz)	Height
1	1.01	403.4	0.1362	19	3.29	1316.6	0.0595	37	3.71	1483.3	0.6579	55	7.47	2984.9	0.3557
2	1.03	410.3	0.2196	20	3.31	1323.4	0.1279	38	3.78	1512.1	0.3880	56	7.48	2989.0	0.3561
3	1.04	415.3	0.2261	21	3.32	1328.0	0.1466	39	3.82	1527.2	0.2083	57	7.49	2993.1	0.4379
4	1.05	421.7	0.1117	22	3.33	1330.3	0.1352	40	4.42	1769.0	0.1790	58	7.50	2998.2	0.1423
5	1.74	696.1	0.0798	23	3.34	1335.3	0.1426	41	4.43	1772.7	0.1918	59	7.51	3002.3	0.1279
6	1.78	710.2	0.4356	24	3.36	1342.2	0.0738	42	6.15	2460.5	0.2468	60	7.52	3006.4	0.1284
7	2.12	845.8	0.3775	25	3.38	1350.9	0.0617	43	6.17	2466.0	0.2705	61	7.61	3043.5	0.1914
8	2.13	849.9	0.4105	26	3.39	1356.0	0.0669	44	6.22	2486.2	0.2234	62	7.62	3047.2	0.2223
9	2.20	881.1	0.9041	27	3.40	1357.8	0.0668	45	6.23	2491.2	0.2139	63	7.65	3060.0	0.1487
10	2.21	885.2	1.0000	28	3.41	1362.8	0.0702	46	6.24	2494.4	0.2239	64	7.66	3063.6	0.1783
11	2.45	980.4	0.7185	29	3.43	1369.7	0.0650	47	7.18	2869.0	0.1538	65	7.69	3076.0	0.3922
12	2.49	996.0	0.5723	30	3.48	1391.2	0.1365	48	7.19	2873.1	0.1690	66	7.70	3080.1	0.2867
13	2.50	1000.1	0.6556	31	3.51	1402.2	0.1586	49	7.22	2885.5	0.1866	67	7.71	3083.8	0.2885
14	2.80	1117.8	0.2101	32	3.56	1423.7	0.3105	50	7.23	2889.6	0.2074	68	7.87	3147.0	0.1343
15	2.82	1129.3	0.2178	33	3.59	1434.7	0.4039	51	7.30	2917.1	0.3059	69	7.88	3151.6	0.1486
16	2.90	1160.9	0.1916	34	3.62	1448.5	0.3603	52	7.31	2921.2	0.3200	70	7.89	3155.2	0.1286
17	2.92	1168.7	0.1662	35	3.67	1469.1	0.5356	53	7.32	2927.2	0.2861	71	7.90	3158.9	0.1035
18	3.17	1268.9	0.3053	36	3.70	1478.7	0.6851	54	7.35	2937.2	0.1278				

No.	Annotation	(ppm)	No.	Annotation	(ppm)
1	31ax,31eq	[1.72 .. 1.85]	8	27	[6.12 .. 6.20]
2	21	[2.44 .. 2.46]	9	25	[6.21 .. 6.29]
3	16,16,16	[2.48 .. 2.55]	10	6	[7.16 .. 7.25]
4	20ax	[2.74 .. 2.86]	11	26	[7.26 .. 7.37]
5	20eq	[2.87 .. 2.97]	12	10,12	[7.42 .. 7.53]
6	29	[3.12 .. 3.21]	13	7	[7.60 .. 7.65]
7	22ax,22eq,30ax,30eq	[3.40 .. 4.01]	14	9,13	[7.67 .. 7.74]

No.	(ppm)	Value	Absolute Value	No.	(ppm)	Value	Absolute Value
1	[7.65 .. 7.67]	2.258	1.86341e+0	11	[2.86 .. 2.99]	8.995	7.42319e+0
2	[4.38 .. 4.48]	3.282	2.70873e+0	12	[2.74 .. 2.85]	9.026	7.44834e+0
3	[7.82 .. 7.97]	4.067	3.35648e+0	13	[7.67 .. 7.79]	10.562	8.71659e+0
4	[7.58 .. 7.64]	4.137	3.41433e+0	14	[3.06 .. 3.23]	11.113	9.17089e+0
5	[7.12 .. 7.25]	6.615	5.45876e+0	15	[2.47 .. 2.55]	15.677	1.29373e+1
6	[3.25 .. 3.37]	7.179	5.92481e+0	16	[7.43 .. 7.56]	16.137	1.33170e+1
7	[6.20 .. 6.32]	8.086	6.67284e+0	17	[7.26 .. 7.40]	17.177	1.41750e+1
8	[2.01 .. 2.15]	8.120	6.70070e+0	18	[1.65 .. 1.87]	17.733	1.46340e+1
9	[0.90 .. 1.12]	8.178	6.74891e+0	19	[2.17 .. 2.30]	19.694	1.62522e+1
10	[6.08 .. 6.20]	8.792	7.25539e+0	20	[3.39 .. 4.13]	127.654	1.05347e+2

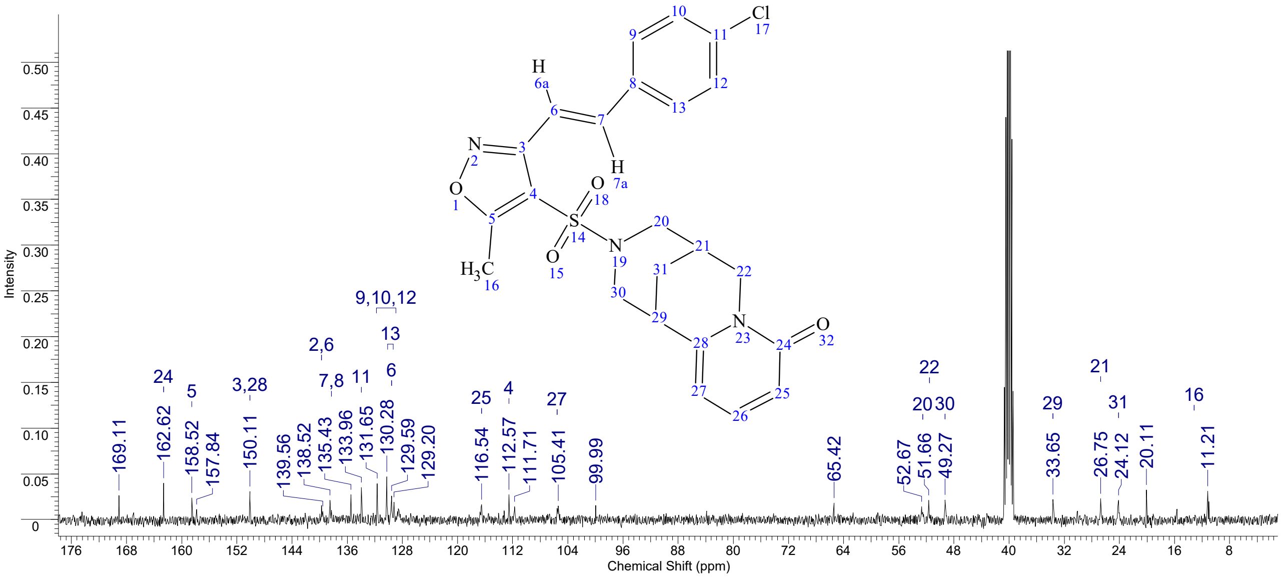

No.	(ppm)	(Hz)	Height	No.	(ppm)	(Hz)	Height
1	11.21	1127.3	0.0309	15	129.20	12988.3	0.0188
2	20.11	2021.5	0.0324	16	129.59	13026.7	0.0260
3	24.12	2425.1	0.0210	17	130.28	13096.6	0.0467
4	26.75	2688.6	0.0227	18	131.65	13233.7	0.0392
5	33.65	3382.6	0.0218	19	133.96	13466.6	0.0349
6	49.27	4952.5	0.0216	20	135.43	13614.2	0.0271
7	51.66	5193.1	0.0209	21	138.52	13924.7	0.0212
8	52.67	5294.7	0.0141	22	139.56	14029.2	0.0088
9	65.42	6576.2	0.0179	23	150.11	15090.2	0.0305
10	99.99	10051.6	0.0153	24	157.84	15866.6	0.0111
11	105.41	10596.0	0.0150	25	158.52	15935.6	0.0235
12	111.71	11229.5	0.0143	26	162.62	16347.7	0.0399
13	112.57	11315.8	0.0271	27	169.11	16999.5	0.0264
14	116.54	11715.5	0.0164				

No.	Annotation	(ppm)	No.	Annotation	(ppm)
1	16	13.14	11	9,10,12	[128.92 .. 131.75]
2	31	24.12	12	13	[129.32 .. 130.13]
3	21	26.75	13	6	129.59
4	29	33.65	14	11	133.96
5	30	49.27	15	7,8	138.31
6	22	51.52	16	2,6	139.70
7	20	52.54	17	3,28	150.11
8	27	105.57	18	5	158.52
9	4	112.57	19	24	162.62
10	25	116.54			

Figure S6. ^{1}H (399.78 MHz, DMSO-d6) and ^{13}C (100.53 MHz, DMSO-d6) NMR Spectra of **3**

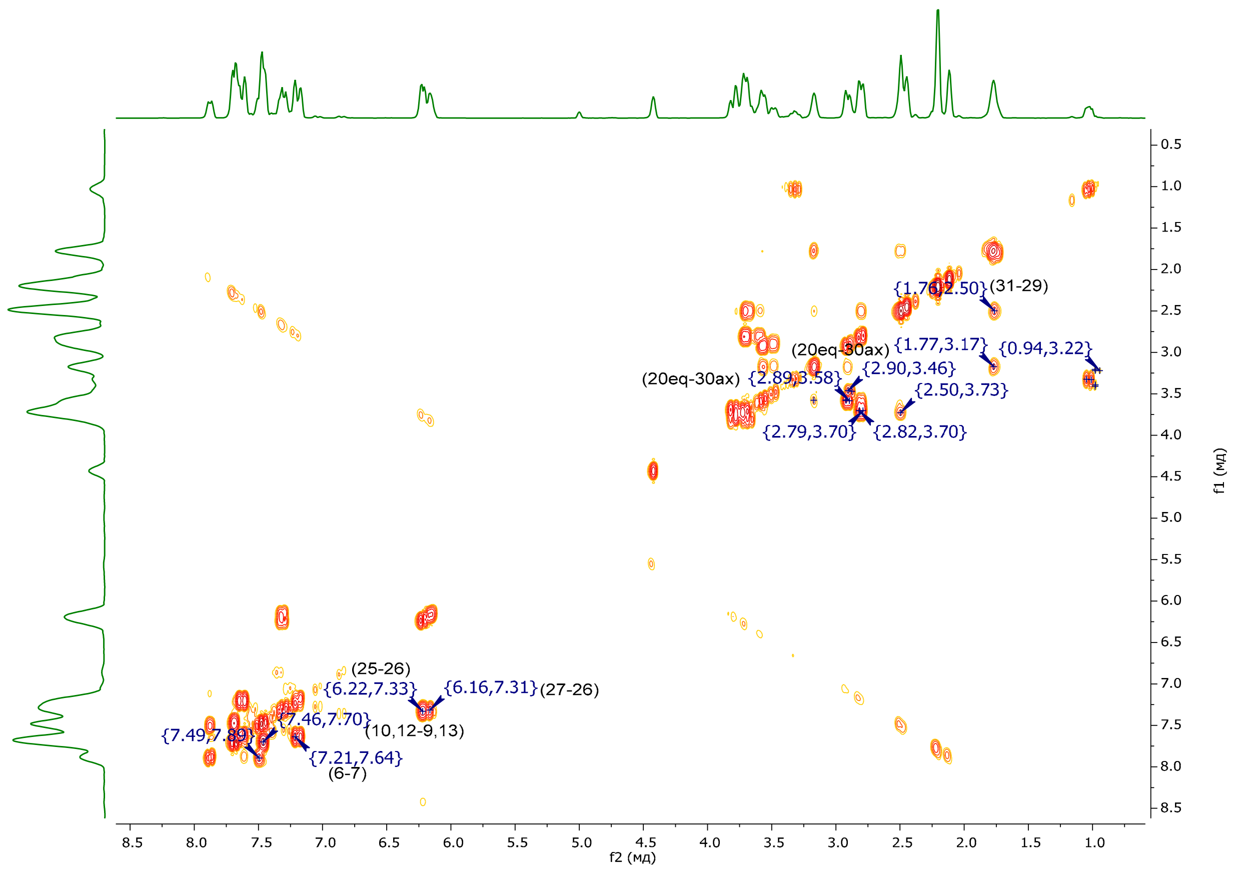

Figure S7. Cosy of **3** in_DMSO

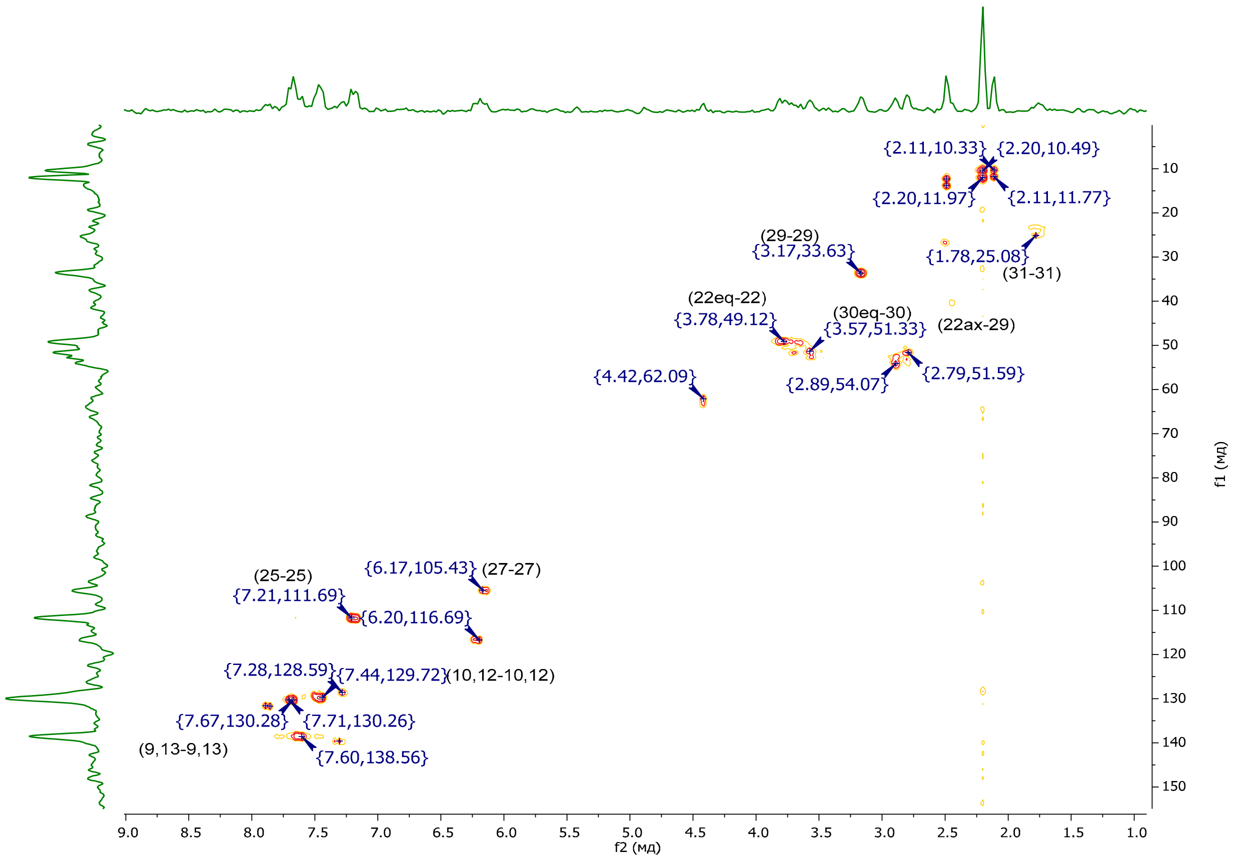

Figure S8 Hmqc of **3** in_DMSO

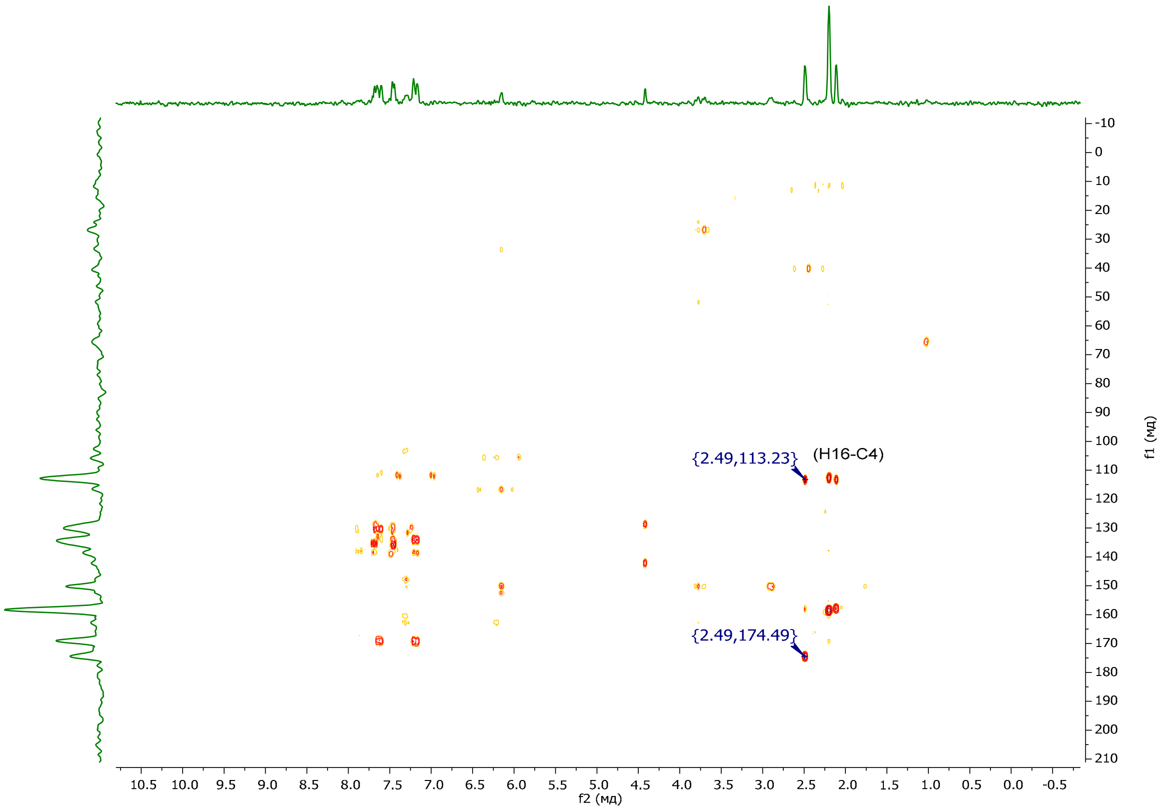

Figure S9. Hmbc of **3** in_DMSO

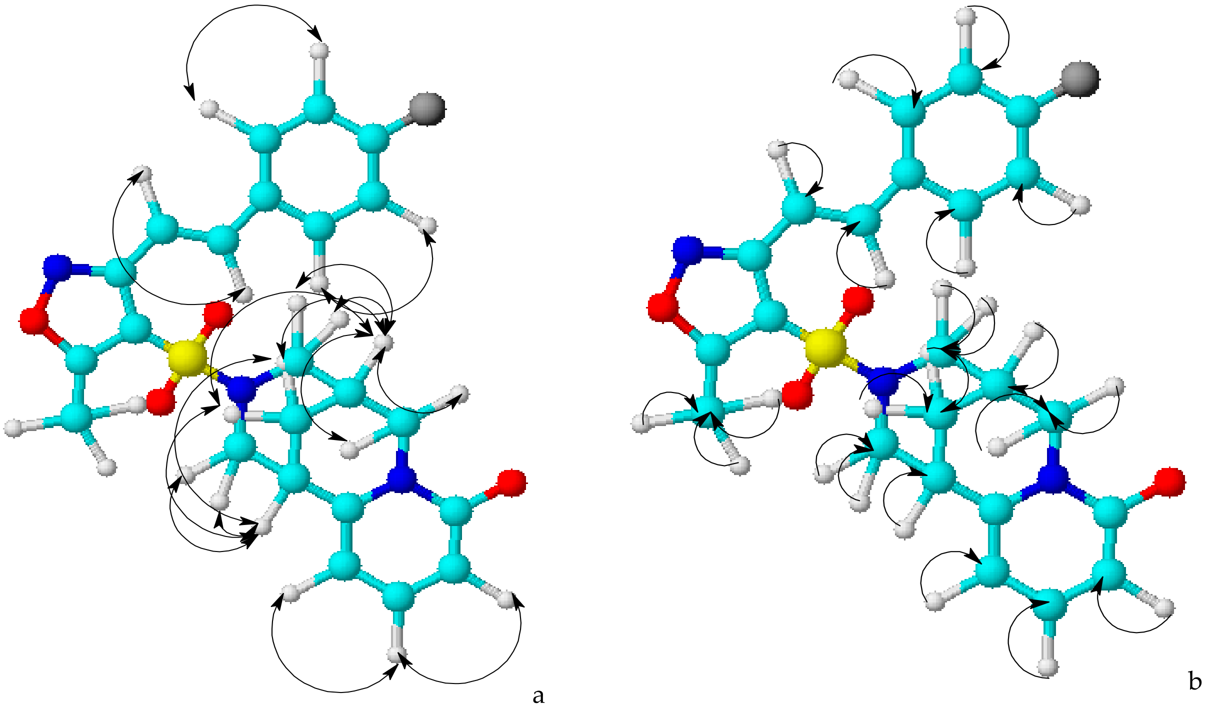

Figure S10. The structural correlations in COSY (a) and HMQC (b) spectra of compound **3**

The 1H-1H COSY spectra of compound 3 have been as follows: has demonstrated the spin-spin correlations through three proton bonds of near methylene-methylene, methine-methylene and methine-methine groups of H31-H29 (1.76, 3.16 and 3.16, 1.76), H20eq-H30eq (2.90, 3.58 and 3.58, 2.90), H20eq-H30ax (2.88, 3.49 and 3.49, 2.88), H25-H26 (6.21, 7.33 and 7.33, 6.21), H27-H26 (6.16, 7.32 and 7.32, 6.16), H6-H7 (7.20, 7.63 and 7.63, 7.20) and H10,12-H9,13 (7.46, 7.70 and 7.70, 7.46) ppm.

Hetero-nuclear couplings of protons with carbon atoms through a single bond have been established by 1H-13C HMQC spectroscopy for the following pairs in a compound: H31-C31 (1.76, 24.48), H21-C21 (2.8, 26.78), H16-C16 (2.49, 13.01), H20ax-C20 (2.78, 52.03), H20eq-C20 (2.90, 51.61), H22ax-C22 (3.53, 51.61), H22eq-C22 (3.70, 51.61), H29-C29 (3.16, 33.87), H30ax-C30 (3.49, 49.81), H30eq-C30 (3.60, 50.20), H26-C26 (7.31, 140.08), H7-C7 (7.63, 138.62), H6-C6 (7.20, 128.82), H25-C25 (6.22, 117.13), H27-C27 (6.16, 105.65), H10,12-C10,12 (7.45, 130.07) and H9,13-C9,13 (7.69, 130.28) ppm.

Hetero-nuclear couplings of protons with carbon atoms through two or more bonds have been determined by 1H-13C HMBC spectroscopy for the following pairs in a compound: H16-C4 (2.49, 113.39) ppm.

Copies of MS Spectra of Products

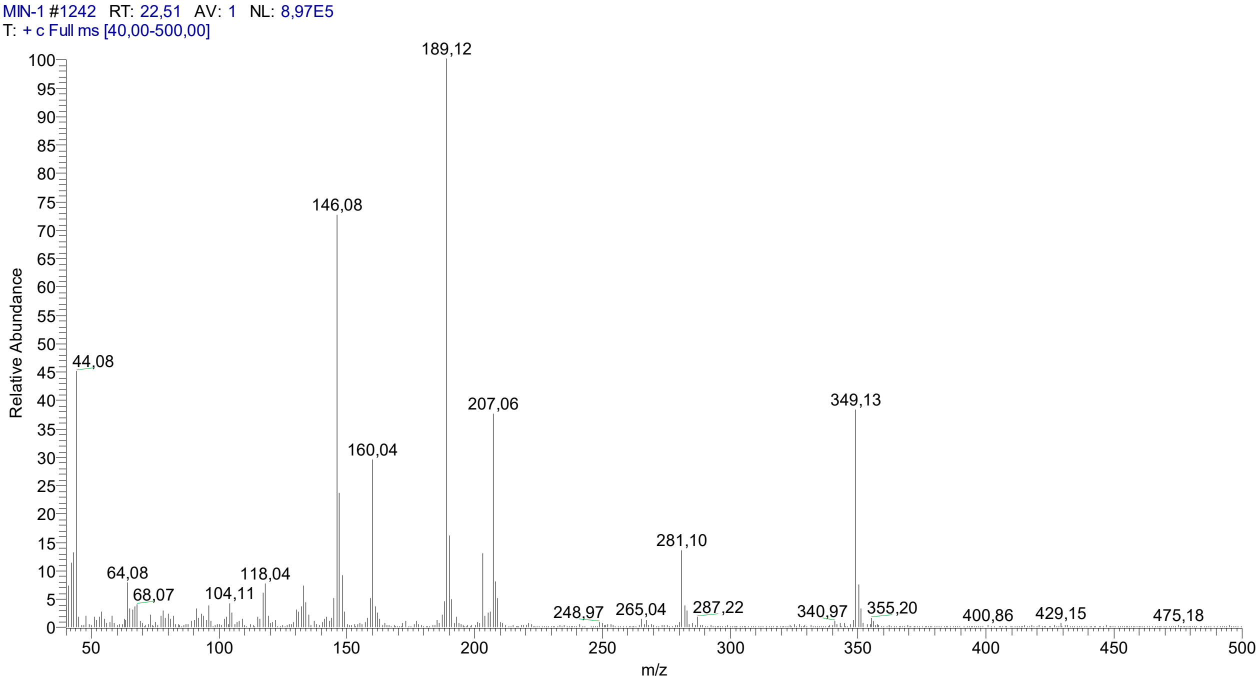

Figure S11. Mass spectrum of **2**

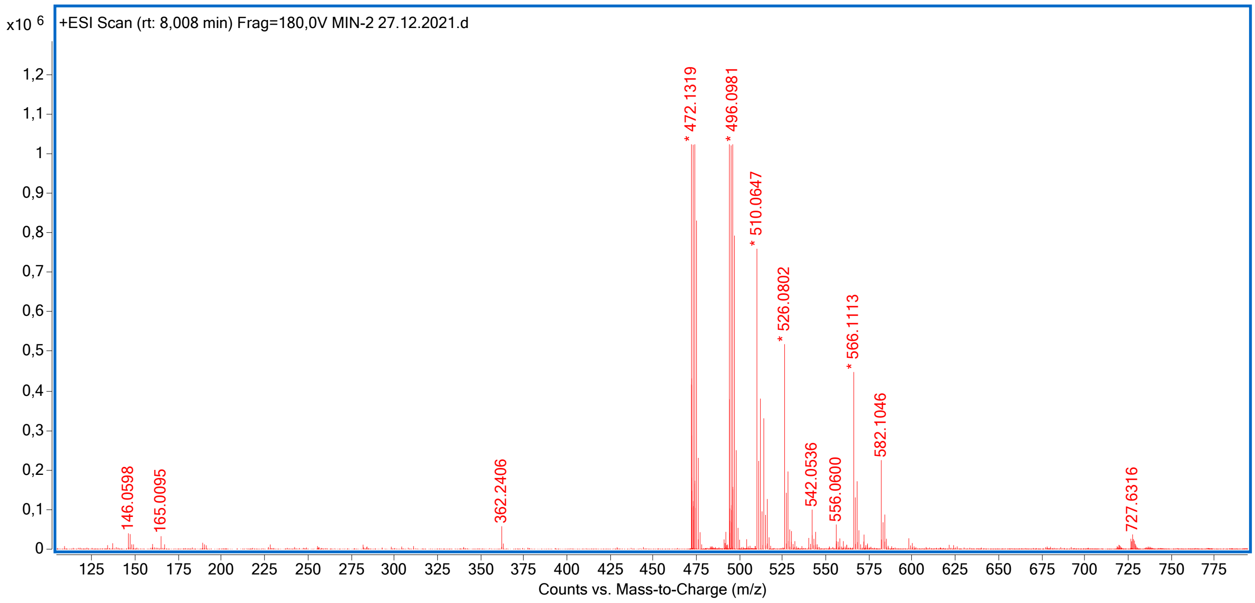

Figure S12. Mass spectrum of **3**

X-Ray Structural Study of Product 2

The cell parameters and intensities of 8569 reflections (3147 independent, Rint = 0.029) have been measured on Bruker KARRA APEX2 CCD (MoKα) diffractometer, graphite monochromator, φ,θ-scanning, 2.721°< θ < 25.990°) at a temperature of 296 K.

Crystals 2 has been monoclinic, a space group $P2_1$, a = 6.6291(3) Å, b = 8.9064(5) Å, c = 13.9604(7) Å, β = 98.093(2)°, V= 816.03(7) A3, Z= 2 (C16H19N3O4S), M = 349.40 g/mol, dcalc.= 1.422 g/cm3, μ = 0.225 mm-1. The array of measured intensities has been processed. Ab-sorption corrections have been performed using the SAINT [24] and SADABS pro-grams included in APEX2 software package (multiscan, Tmin = 0.871, Tmax = 0.904).

Structure has been decoded with a direct method. Positions of the non-hydrogen atoms have been clarified in the anisotropic approximation by the full matrix MNA. Hydrogen atoms have been placed in the geometrically calculated positions. Their posi-tions have been refined in the isotropic approximation with the fixed positional and thermal parameters (a "rider" model). Calculations have used 2944 independent reflections with I>2σ(I). The number of the refined parameters has been 219. Final divergence factors have been R1 = 0.0317, wR2 = 0.0749 for reflections with I>2σ(I), R1= 0.0359, wR2 = 0.0784 for all reflections, GOOF = 0.990. The residual density peaks: Δρ = 0.184 e/A3 and -0.204 e/A3. Structure has been decoded and refined with using the programs of SHELXT-2014/5 and SHELXL-2018/3. The X-ray diffraction data in the form of a CIF file has been deposited in the Cambridge Crystallographic Data Center (CCDC 2168324).

Table 1. Bond lengths (d, Å) in molecule **2**

Bond	d	Bond	d
S(1)-O(3)	1.424(2)	C(9)-C(13)	1.523(4)
S(1)-O(2)	1.427(2)	C(9)-H(9A)	0.9800
S(1)-N(12)	1.634(2)	C(10)-H(10A)	0.9700
S(1)-C(4')	1.749(3)	C(10)-H(10B)	0.9700
O(1)-C(2)	1.231(3)	C(11)-N(12)	1.472(4)
N(1)-C(6)	1.372(3)	C(11)-H(11A)	0.9700
N(1)-C(2)	1.400(3)	C(11)-H(11B)	0.9700
N(1)-C(10)	1.487(3)	N(12)-C(13)	1.484(3)
C(2)-C(3)	1.427(4)	C(13)-H(13A)	0.9700
C(3)-C(4)	1.348(5)	C(13)-H(13B)	0.9700
C(3)-H(3B)	0.9300	O(1')-C(5')	1.334(4)
C(4)-C(5)	1.396(4)	O(1')-N(2')	1.418(4)
C(4)-H(4A)	0.9300	N(2')-C(3')	1.304(4)
C(5)-C(6)	1.362(4)	C(3')-C(4')	1.412(4)
C(5)-H(5A)	0.9300	C(3')-C(6')	1.475(6)
C(6)-C(7)	1.500(4)	C(4')-C(5')	1.372(4)
C(7)-C(8)	1.526(4)	C(5')-C(7')	1.480(5)
C(7)-C(11)	1.532(4)	C(6')-H(6'A)	0.9600
C(7)-H(7A)	0.9800	C(6')-H(6'B)	0.9600
C(8)-C(9)	1.515(5)	C(6')-H(6'C)	0.9600
C(8)-H(8A)	0.9700	C(7')-H(7'A)	0.9600
C(8)-H(8B)	0.9700	C(7')-H(7'B)	0.9600
C(9)-C(10)	1.512(4)	C(7')-H(7'C)	0.9600

Table 2. Bond angles (ω, deg.) in molecule **2**

Angle	ω	Angle	ω
O(3)-S(1)-O(2)	120.01(14)	N(1)-C(10)-H(10A)	108.6
O(3)-S(1)-N(12)	107.74(13)	C(9)-C(10)-H(10A)	108.6
O(2)-S(1)-N(12)	107.29(13)	N(1)-C(10)-H(10B)	108.6
O(3)-S(1)-C(4')	106.65(14)	C(9)-C(10)-H(10B)	108.6
O(2)-S(1)-C(4')	107.25(14)	H(10A)-C(10)-H(10B)	107.6
N(12)-S(1)-C(4')	107.31(12)	N(12)-C(11)-C(7)	108.9(2)
C(6)-N(1)-C(2)	123.0(2)	N(12)-C(11)-H(11A)	109.9
C(6)-N(1)-C(10)	123.5(2)	C(7)-C(11)-H(11A)	109.9
C(2)-N(1)-C(10)	113.4(2)	N(12)-C(11)-H(11B)	109.9
O(1)-C(2)-N(1)	119.6(3)	C(7)-C(11)-H(11B)	109.9
O(1)-C(2)-C(3)	124.8(3)	H(11A)-C(11)-H(11B)	108.3
N(1)-C(2)-C(3)	115.6(3)	C(11)-N(12)-C(13)	113.4(2)
C(4)-C(3)-C(2)	121.2(3)	C(11)-N(12)-S(1)	117.54(17)
C(4)-C(3)-H(3B)	119.4	C(13)-N(12)-S(1)	115.02(19)
C(2)-C(3)-H(3B)	119.4	N(12)-C(13)-C(9)	109.5(2)
C(3)-C(4)-C(5)	120.8(3)	N(12)-C(13)-H(13A)	109.8
C(3)-C(4)-H(4A)	119.6	C(9)-C(13)-H(13A)	109.8
C(5)-C(4)-H(4A)	119.6	N(12)-C(13)-H(13B)	109.8
C(6)-C(5)-C(4)	120.1(3)	C(9)-C(13)-H(13B)	109.8
C(6)-C(5)-H(5A)	120.0	H(13A)-C(13)-H(13B)	108.2
C(4)-C(5)-H(5A)	120.0	C(5')-O(1')-N(2')	108.7(2)
C(5)-C(6)-N(1)	119.3(2)	C(3')-N(2')-O(1')	106.7(3)
C(5)-C(6)-C(7)	122.0(2)	N(2')-C(3')-C(4')	110.1(3)
N(1)-C(6)-C(7)	118.7(2)	N(2')-C(3')-C(6')	118.5(3)
C(6)-C(7)-C(8)	110.9(2)	C(4')-C(3')-C(6')	131.4(3)
C(6)-C(7)-C(11)	111.1(2)	C(5')-C(4')-C(3')	105.9(3)
C(8)-C(7)-C(11)	109.8(2)	C(5')-C(4')-S(1)	126.7(2)
C(6)-C(7)-H(7A)	108.3	C(3')-C(4')-S(1)	127.4(2)
C(8)-C(7)-H(7A)	108.3	O(1')-C(5')-C(4')	108.6(3)
C(11)-C(7)-H(7A)	108.3	O(1')-C(5')-C(7')	117.0(3)
C(9)-C(8)-C(7)	106.5(2)	C(4')-C(5')-C(7')	134.4(3)
C(9)-C(8)-H(8A)	110.4	C(3')-C(6')-H(6'A)	109.5
C(7)-C(8)-H(8A)	110.4	C(3')-C(6')-H(6'B)	109.5
C(9)-C(8)-H(8B)	110.4	H(6'A)-C(6')-H(6'B)	109.5
C(7)-C(8)-H(8B)	110.4	C(3')-C(6')-H(6'C)	109.5
H(8A)-C(8)-H(8B)	108.6	H(6'A)-C(6')-H(6'C)	109.5
C(10)-C(9)-C(8)	111.1(2)	H(6'B)-C(6')-H(6'C)	109.5
C(10)-C(9)-C(13)	112.2(2)	C(5')-C(7')-H(7'A)	109.5
C(8)-C(9)-C(13)	110.4(2)	C(5')-C(7')-H(7'B)	109.5
C(10)-C(9)-H(9A)	107.6	H(7'A)-C(7')-H(7'B)	109.5
C(8)-C(9)-H(9A)	107.6	C(5')-C(7')-H(7'C)	109.5
C(13)-C(9)-H(9A)	107.6	H(7'A)-C(7')-H(7'C)	109.5
N(1)-C(10)-C(9)	114.4(2)	H(7'B)-C(7')-H(7'C)	109.5

Table 3. Torsion angles (τ, deg.) in molecule **2**

Angle	τ	Angle	τ
C(6)-N(1)-C(2)-O(1)	179.4(2)	O(3)-S(1)-N(12)-C(11)	168.7(2)
C(10)-N(1)-C(2)-O(1)	1.6(4)	O(2)-S(1)-N(12)-C(11)	38.1(2)
C(6)-N(1)-C(2)-C(3)	-0.9(4)	C(4')-S(1)-N(12)-C(11)	-76.8(2)
C(10)-N(1)-C(2)-C(3)	-178.7(2)	O(3)-S(1)-N(12)-C(13)	-53.9(2)
O(1)-C(2)-C(3)-C(4)	-178.3(3)	O(2)-S(1)-N(12)-C(13)	175.6(2)
N(1)-C(2)-C(3)-C(4)	2.0(4)	C(4')-S(1)-N(12)-C(13)	60.6(2)
C(2)-C(3)-C(4)-C(5)	-1.2(5)	C(11)-N(12)-C(13)-C(9)	-56.2(3)
C(3)-C(4)-C(5)-C(6)	-0.9(5)	S(1)-N(12)-C(13)-C(9)	164.5(2)
C(4)-C(5)-C(6)-N(1)	1.9(4)	C(10)-C(9)-C(13)-N(12)	-66.1(3)
C(4)-C(5)-C(6)-C(7)	-177.7(3)	C(8)-C(9)-C(13)-N(12)	58.4(3)
C(2)-N(1)-C(6)-C(5)	-1.1(4)	C(5')-O(1')-N(2')-C(3')	-0.2(4)
C(10)-N(1)-C(6)-C(5)	176.5(3)	O(1')-N(2')-C(3')-C(4')	0.4(4)
C(2)-N(1)-C(6)-C(7)	178.6(2)	O(1')-N(2')-C(3')-C(6')	178.5(4)
C(10)-N(1)-C(6)-C(7)	-3.8(3)	N(2')-C(3')-C(4')-C(5')	-0.4(4)
C(5)-C(6)-C(7)-C(8)	-148.2(3)	C(6')-C(3')-C(4')-C(5')	-178.2(4)
N(1)-C(6)-C(7)-C(8)	32.1(3)	N(2')-C(3')-C(4')-S(1)	178.4(2)
C(5)-C(6)-C(7)-C(11)	89.3(3)	C(6')-C(3')-C(4')-S(1)	0.6(6)
N(1)-C(6)-C(7)-C(11)	-90.3(3)	O(3)-S(1)-C(4')-C(5')	29.7(3)
C(6)-C(7)-C(8)-C(9)	-60.9(3)	O(2)-S(1)-C(4')-C(5')	159.4(3)
C(11)-C(7)-C(8)-C(9)	62.3(3)	N(12)-S(1)-C(4')-C(5')	-85.6(3)
C(7)-C(8)-C(9)-C(10)	63.5(3)	O(3)-S(1)-C(4')-C(3')	-148.9(3)
C(7)-C(8)-C(9)-C(13)	-61.6(3)	O(2)-S(1)-C(4')-C(3')	-19.1(3)
C(6)-N(1)-C(10)-C(9)	5.7(4)	N(12)-S(1)-C(4')-C(3')	95.9(3)
C(2)-N(1)-C(10)-C(9)	-176.5(2)	N(2')-O(1')-C(5')-C(4')	0.0(3)
C(8)-C(9)-C(10)-N(1)	-36.3(3)	N(2')-O(1')-C(5')-C(7')	-179.3(3)
C(13)-C(9)-C(10)-N(1)	87.8(3)	C(3')-C(4')-C(5')-O(1')	0.3(3)
C(6)-C(7)-C(11)-N(12)	63.2(3)	S(1)-C(4')-C(5')-O(1')	-178.6(2)
C(8)-C(7)-C(11)-N(12)	-59.9(3)	C(3')-C(4')-C(5')-C(7')	179.3(4)
C(7)-C(11)-N(12)-C(13)	56.9(3)	S(1)-C(4')-C(5')-C(7')	0.5(5)
C(7)-C(11)-N(12)-S(1)	-164.97(18)		
